# Identification of alternative splicing associated with clinical features: from pan-cancers to genitourinary tumors

**DOI:** 10.3389/fonc.2023.1249932

**Published:** 2023-09-25

**Authors:** Chen Duan, Yangjun Zhang, Lu Li, Kai Liu, Xiangyang Yao, Xiaoliang Wu, Bo Li, Xiongmin Mao, Huahui Wu, Haoran Liu, Jin Zeng, Sheng Li, Yan Gong, Zhiquan Hu, Hua Xu

**Affiliations:** ^1^ Department of Urology, Tongji Hospital, Tongji Medical College, Huazhong University of Science and Technology, Wuhan, Hubei, China; ^2^ Tumor Precision Diagnosis and Treatment Technology and Translational Medicine, Hubei Engineering Research Center, Zhongnan Hospital of Wuhan University, Wuhan, Hubei, China; ^3^ Department of Urology, Zhongnan Hospital of Wuhan University, Wuhan, Hubei, China; ^4^ Department of Radiation Oncology, Sichuan Cancer Hospital and Institute, Sichuan Cancer Center, School of Medicine, University of Electronic Science and Technology of China, Chengdu, Sichuan, China; ^5^ Department of Urology, Stanford University School of Medicine, Stanford, CA, United States; ^6^ Department of Urology, The First Affiliated Hospital of Nanchang University, Nanchang, Jiangxi, China; ^7^ Taikang Center for Life and Medical Sciences, Wuhan University, Wuhan, Hubei, China

**Keywords:** alternative splicing, pan-cancers, tumor heterogeneity, clinical features, genitourinary tumors

## Abstract

**Background:**

Alternative splicing events (ASEs) are vital causes of tumor heterogeneity in genitourinary tumors and many other cancers. However, the clinicopathological relevance of ASEs in cancers has not yet been comprehensively characterized.

**Methods:**

By analyzing splicing data from the TCGA SpliceSeq database and phenotype data for all TCGA samples from the UCSC Xena database, we identified differential clinical feature-related ASEs in 33 tumors. CIBERSORT immune cell infiltration data from the TIMER2.0 database were used for differential clinical feature-related immune cell infiltration analysis. Gene function enrichment analysis was used to analyze the gene function of ASEs related to different clinical features in tumors. To reveal the regulatory mechanisms of ASEs, we integrated race-related ASEs and splicing quantitative trait loci (sQTLs) data in kidney renal clear cell carcinoma (KIRC) to comprehensively assess the impact of SNPs on ASEs. In addition, we predicted regulatory RNA binding proteins in bladder urothelial carcinoma (BLCA) based on the enrichment of motifs around alternative exons for ASEs.

**Results:**

Alternative splicing differences were systematically analyzed between different groups of 58 clinical features in 33 cancers, and 30 clinical features in 24 cancer types were identified to be associated with more than 50 ASEs individually. The types of immune cell infiltration were found to be significantly different between subgroups of primary diagnosis and disease type. After integrating ASEs with sQTLs data, we found that 63 (58.9%) of the race-related ASEs were significantly SNP-correlated ASEs in KIRC. Gene function enrichment analyses showed that metastasis-related ASEs in KIRC mainly enriched Rho GTPase signaling pathways. Among those ASEs associated with metastasis, alternative splicing of GIT2 and TUBB3 might play key roles in tumor metastasis in KIRC patients. Finally, we identified several RNA binding proteins such as PCBP2, SNRNP70, and HuR, which might contribute to splicing differences between different groups of neoplasm grade in BLCA.

**Conclusion:**

We demonstrated the significant clinical relevance of ASEs in multiple cancer types. Furthermore, we identified and validated alternative splicing of TUBB3 and RNA binding proteins such as PCBP2 as critical regulators in the progression of urogenital cancers.

## Introduction

As the second leading cause of death worldwide, cancer kills several millions of people per year, and this number has been increasing for decades (1, 2). Nowadays, multiple cancer therapies are available and greatly improve patient outcomes, such as surgical procedures, chemoradiotherapy, immunotherapy, targeted therapy, and comprehensive treatment. Although exciting progress has been made in cancer therapy, the extensive heterogeneity and complexity of tumors pose tremendous challenges to the treatment of tumors. This heterogeneity underlies the emergence of resistance to cancer therapies to a great extent. As patients with tumors of the same histological type often respond differently to the same treatment (3), personalized treatment is the future direction, which requires a deeper understanding of tumor heterogeneity and its clinical relevance.

Tumor heterogeneity is now a broad concept that means both the inter-patient heterogeneity resulting from genetic variation or environment factors and the intratumoral heterogeneity manifested as genetically diverse subpopulations or dynamic genetic variations over time of an individual tumor (4). Advances in sequencing technologies and computational methods enable the uncovering of tumor heterogeneity and its clinical relevance (5–7). Van Allen et al. generated a database of gene alterations with clinical implications for cancer patients by using whole exome sequencing and applied it to clinical decision-making (8). By using targeted deep sequencing, Burkhardt et al. revealed age dependency of mutations in Burkitt lymphoma in children and adolescents (9). Drews et al. illuminated the relationship between chromosomal instability and clinical phenotypes in cancers and predicted platinum sensitivity of ovarian cancer by integrating impaired homologous recombination signatures (10). Previous studies also investigated the roles of post-transcriptional mechanisms in tumor heterogeneity. Han et al. identified clinically relevant adenosine-to-inosine RNA editing events (11). Several researchers focused on the methylation of N^6^ adenosine (m^6^A), and found that the landscape of m^6^A regulators was related to cancer progression (12–14). All these studies characterized the profiles of molecular alterations related to clinical characteristics across cancers and guided individualized treatment of cancers. However, the specific mechanisms of how post-transcriptional regulation affect tumor heterogeneity and tumorigenesis still need further study.

Alternative splicing (AS) is a vital cause of tumor heterogeneity. As a common post-transcriptional regulation, AS causes distinct transcript isoforms and protein variants (15). Aberrant AS, which is significantly increased in malignant tissues, reshapes the transcriptome and proteome, influencing signaling pathways related to cellular homeostasis, cell proliferation, and differentiation (16, 17). For instance, the CD44 splice isoform promotes epithelial-mesenchymal transition across cancers (18–21). AS is precisely regulated by RNA-binding proteins (RBPs), especially splicing factors including serine/arginine-rich (SR) proteins and heterogeneous nuclear ribonucleoproteins (hnRNPs) (22). SR proteins and hnRNPs regulate the recruitment and function of spliceosomes, thereby promoting or repressing splicing. Mutation or dysfunction of splicing factors can cause the generation of abnormal isoforms associated with the occurrence and progression of tumors (23). Numerous studies demonstrated that the expression of splicing factors varied in normal tissues and cancers, while altered expression or activity of splicing factors could contribute to cancer progression. The mutation of splicing factor U2AF1 and relative aberrant AS events were identified in multiple cancer types (24), while splicing factor SF3B1 was suggested to be a therapeutic target for breast cancer patients (25). Therefore, clinically relevant aberrant AS events (ASEs) and specific splicing factors can be promising targets for cancer therapy.

To comprehensively reveal the clinically relevant differential ASEs involved in tumorigenesis, progression, and prognosis of multiple cancers, we integrally analyzed mRNA splicing data in 31 human cancers, identified thousands of tumor-specific and survival-associated ASEs, and created a website tool OncoSplicing for the exploration and visualization of clinical relevant ASEs (26, 27). In this study, we analyzed multiple clinical indicator-relevant differential ASEs in 33 human cancers based on the OncoSplicing database and explored changes in splicing factors expression. Furthermore, we identified alternative splicing of GIT2 and TUBB3 and RNA binding proteins such as PCBP2 as critical regulators in the progression of urogenital cancers. Together, we believe that our work will help to further reveal the relationship between AS and clinicopathological features in cancers, and also to provide more available molecular targets for cancer therapy by regulating clinically relevant AS and splicing factors.

## Materials and methods

### Data acquisition and pre-processing

Splicing data for all splicing events of 33 tumors were downloaded from the TCGA SpliceSeq database (http://projects.insilico.us.com/TCGASpliceSeq/PSIdownload.jsp), with the parameter percent-samples-with-values (PCT) set to no less than 75%. This contains splicing events belonging to 7 splicing types, including alternative acceptor sites (AA), alternative donor sites (AD), exon skipping (ES), mutually exclusive exons (ME), retained introns (RI), alternate promoter (AP) and alternate terminator (AT).

Phenotype data for all TCGA samples were downloaded in the GDC cohort of each cancer type separately from the UCSC Xena database (https://xenabrowser.net/datapages/). Basic patient information, such as age (full name is “age at initial pathologic diagnosis”), gender, race, and other nonredundant and different clinical features, were manually collected, by which patients were separated into 2 groups for each cancer type. Clinical features with continuous data in each cancer type were grouped by the median cutoff value. For category clinical features, the top 2 groups with the most samples in each cancer type were kept. After integration with splicing data by samples, clinical features in a cancer type were reserved for further analysis only if there were > 20 records per group.

### Identification of clinical feature-related alternative splicing event

Differential alternative splicing analysis was performed between 2 groups for each clinical feature. The P value of the significance of the difference was evaluated using the Wilcoxon rank sum test, and the splicing events with the absolute value of the delta PSI greater than 0.1 and the Benjamini-Hochberg (BH) adjusted P value less than 0.05 were considered as clinical feature related ASEs.

### Identification of clinical feature-related immune cell infiltration

CIBERSORT immune cell infiltration data were obtained from the TIMER2.0 database (http://timer.cistrome.org/). Differential immune cell infiltration analysis was performed between 2 groups for each clinical feature. The P value of the significance of the difference was evaluated using the Wilcoxon rank sum test, and cell types with the absolute delta proportion greater than 3 percent and the Benjamini-Hochberg (BH) adjusted P value less than 0.05 were considered as significant.

### Gene function enrichment analysis

Genes of clinical feature-related ASEs for each interested clinical feature in individual cancer types were submitted to Metascape (http://metascape.org) separately to implement gene function enrichment analysis. The top 10 pathways with significant P values were analyzed and displayed. Plots of survival and differences between 2 groups for ASEs enriched in interesting pathways were performed using the OncoSplicing website (www.oncosplicing.com).

### Structure and genomic location of alternative splicing events

The gene structure file was downloaded from the TCGA SpliceSeq database to obtain the genomic position of each exon. According to the exon composition of a splicing event, the genome locations of the whole event and the start-end positions of the alternative exon are determined, and then the range between 250 nt upstream and downstream of the alternative exon was considered the region that most likely influenced the splicing level of a splicing event.

### Splicing quantitative trait loci analysis

The splicing quantitative trait loci (sQTLs) data of the renal clear cell carcinoma cohort was downloaded from the CancerSplicingQTL database (http://www.cancersplicingqtl-hust.com/#/). Significant SNPs associated with each splicing event were identified and mapped to the genome locations of the splicing event, and only SNPs in the range between 250 nt upstream and downstream of alternative exon were considered meaningful sQTLs.

### Splicing events map to RNA motifs of RBPs

To explore the potential regulator of clinical feature-related splicing events in each cancer type, splicing events were extracted with genome coordination and organized into the format that meets the requirements of rMAPS2 (http://rmaps.cecsresearch.org/) for each splice type separately (28). Splice types with considerable AS events were chosen to perform enrichment analysis of RBP motifs. Clinical feature-related alternative splicing events were separated as up-regulated and down-regulated events by the direction of PSI change. ASEs with delta PSI less than 0.001 and FDR more than 0.9 were set as background control.

### Cell culture and stable transfection

Kidney cancer (OS-RC-2 and 786-O) and bladder cancer (T24 and 5637) cells were obtained from the Shanghai Cell Bank Type Culture Collection Committee and maintained in Department of Biological Repositories, Zhongnan Hospital of Wuhan University (Wuhan, China). Cells used in this study underwent STR authentication. Tumor cells were cultured in McCoy’s 5A (T24) and RPMI-1640 (OS-RC-2, 786-O, and 5637) medium containing 10% FBS (Gibco, USA). The lentiviruses carrying small hairpin RNAs (shRNA) targeting TUBB3, PCBP2, and control shRNA were constructed by Tsingke (Beijing, China). The target sequences were as follows: 5’-CAGTATTTATGGCCTCGTCCT-3’ (shRNA1-TUBB3), 5’-CATCTCTTCAGGCCTGACAAT-3’ (shRNA2-TUBB3), 5’-CCATGATCCATCTGTGTAGTT-3’ (shRNA1-PCBP2) and 5’-CCCACTAATGCCATCTTCAAA-3’ (shRNA2-PCBP2). Lipo2000 (Invitrogen, 11668030, USA) was used to transfect the lentiviral vectors into tumor cells, and the transfection efficiency was confirmed by qPCR.

### qPCR

Total RNAs were extracted from cells by using Trizol reagent (Thermo Fisher Scientific, USA). HiScript III RT SuperMix (Vazyme, R323-01, China) was applied to synthesize cDNA, and ChamQ Universal SYBR qPCR Master Mix (Vazyme, Q711-02/03, China) was used for real-time PCR experiments according to the manufacturer’s instructions. qPCR analysis was performed on QuantStudio Flex system (Applied Biosystem, USA). Primers sequences were as follows: TUBB3, 5′-GACTCCCTTGAACAGGGACAG-3′ (forward), 5′-GGCACGTACTTGTGAGAAGA-3′ (reverse); PCBP2, 5′-TCTGCGTGGTCATGTTGGAG-3′ (forward), 5′-TGCATCCAAACCTGCCCAATA-3′ (reverse); GAPDH, 5′-AATGGGCAGCCGTTAGGAAA-3′ (forward), 5′-GCCCAATACGACCAAATCAGAG-3′ (reverse).

### Cell viability assay

Tumor cell proliferation was detected by CCK-8 assay. Briefly, cells were inoculated into 96-well plates (2×10^3^ cells per well) and cultured in the 5% CO2 incubator at 37°. After mixing with 10 μL CCK-8 reagent (MCE, HY-K0301, USA) for 2 h, absorbance was detected at 450nm with an absorbance reader (Molecular Devices, USA).

### Colony formation assay

Approximately 1000 cells were seeded in a 60 mm petri dish with the complete medium. After 2-3 weeks, cells were fixed for 15 min and stained with crystal violet for 10 min. Visible clones were counted by using ImageJ software.

### Modified Boyden chamber assay

For tumor cell migration, approximately 5×10^4^ cells were suspended in 100 μL serum-free medium and plated in the apical chambers of transwell plates (Corning, USA), and 500 μL complete medium was added in the lower chamber. After 24 h, the migrated cells were fixed and then dyed in crystal violet. The cell number was counted from 6 random fields under an inverted microscope.

### Statistics and visualization

Statistical analysis was performed using R software (4.0.1). Data analysis and visualization tools in the R software include R packages such as ggplot2, ComplexHeatmap, limma, survminner, and venn.diagram. One-way or two-way ANOVA was used to analyze the differences in multiple groups, and a P-value < 0.05 was considered statistically significant.

## Results

### Clinical features in cancers

This study contains 33 TCGA cancers, including Adrenocortical Carcinoma (ACC), Kidney Renal Clear Cell Carcinoma (KIRC), Prostate Adenocarcinoma (PRAD), Bladder Urothelial Carcinoma (BLCA), Kidney Renal Papillary Cell Carcinoma (KIRP), Rectum Adenocarcinoma (READ), Breast Invasive Carcinoma (BRCA), Acute Myeloid Leukemia (LAML), Sarcoma (SARC), Cervical Squamous Cell Carcinoma (CESC), Lower Grade Glioma (LGG), Skin Cutaneous Melanoma (SKCM), Cholangiocarcinoma (CHOL), Liver Hepatocellular Carcinoma (LIHC), Stomach Adenocarcinoma (STAD), Colon Adenocarcinoma (COAD), Lung Adenocarcinoma (LUAD), Testicular Germ Cell Tumors (TGCT), Diffuse Large B-cell Lymphoma (DLBC), Lung Squamous Cell Carcinoma (LUSC), Thyroid Carcinoma (THCA), Esophageal Carcinoma (ESCA), Mesothelioma (MESO), Thymoma (THYM), Glioblastoma Multiforme (GBM), Ovarian Serous Cystadenocarcinoma (OV), Uterine Corpus Endometrial Carcinoma (UCEC), Head and Neck Squamous Cell Carcinoma (HNSC), Pancreatic Adenocarcinoma (PAAD), Uterine Carcinosarcoma (UCS), Kidney Chromophobe (KICH), Pheochromocytoma and Paraganglioma (PCPG), Uveal Melanoma (UVM).

**Figure 1:**
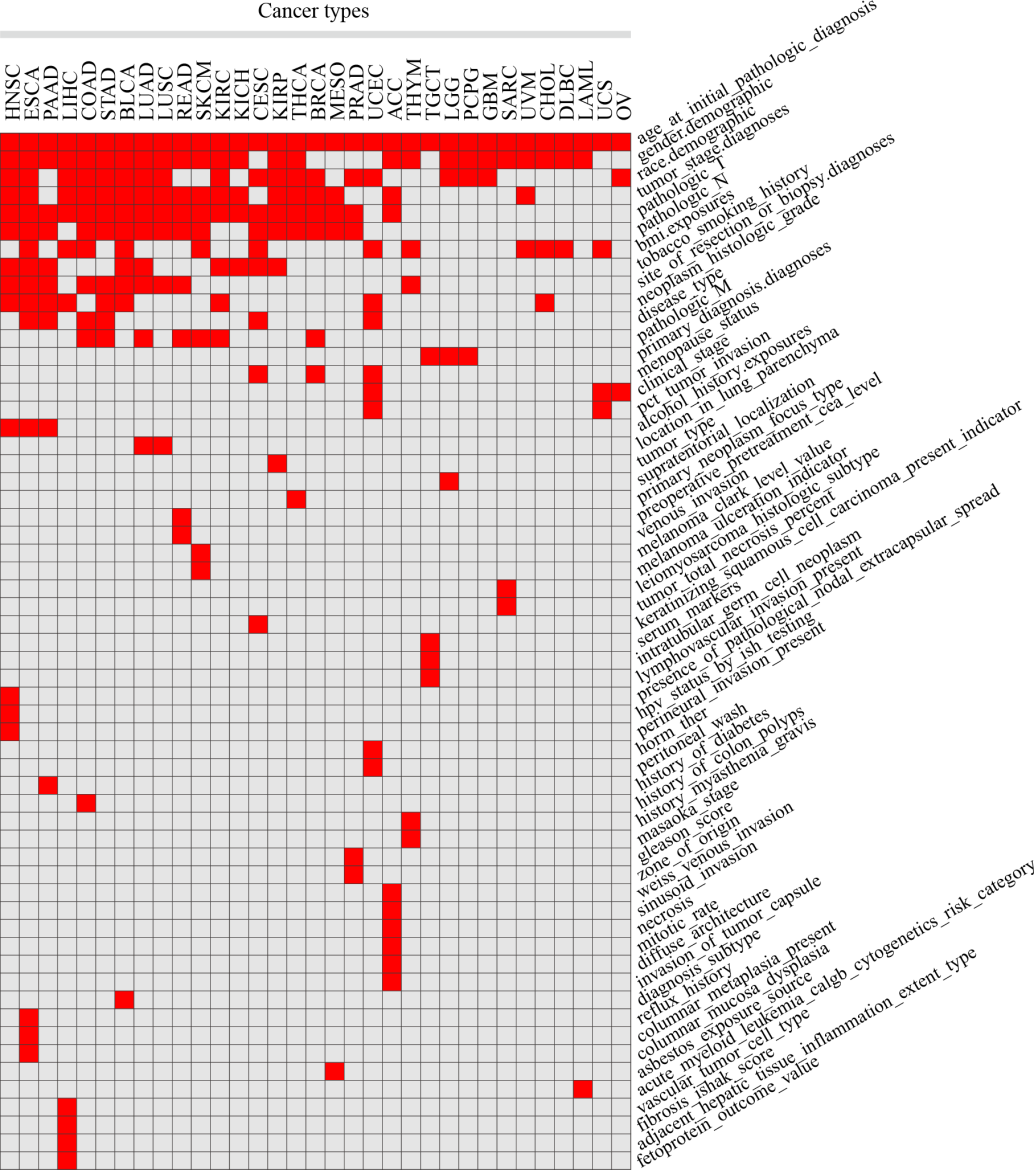
Statistics of different clinical features in 33 TCGA tumors. Red squares indicate that clinical features in the corresponding tumors were included in the analysis, while grey squares indicate that not included.

Clinical features were manually collected, by which patients were separated into 2 groups for each cancer type. After organizing and filtering the phenotype data for each tumor, 58 clinical features were retained in 33 tumors, with an average of 7 in each tumor, 15 in ESCA at most, and 3 in OV at least. Clinical features common in more than 10 tumors included age (n = 33 tumors), gender (n = 25), pathologic T (n = 20), race (n = 19), tumor stage (n = 18), pathologic N (n = 16), BMI (n = 12) and clinicopathological features such as the site of resection or biopsy (n = 10). Other important cancer-specific features such as “history of colon polyps” in COAD, “fetoprotein outcome value” in LIHC, and “acute myeloid leukemia calgb cytogenetics risk category” in LAML were also included (**Figure 1**; **Table S1**).

### Identification of clinical feature-related AS and immune cell infiltration

**Figure 2:**
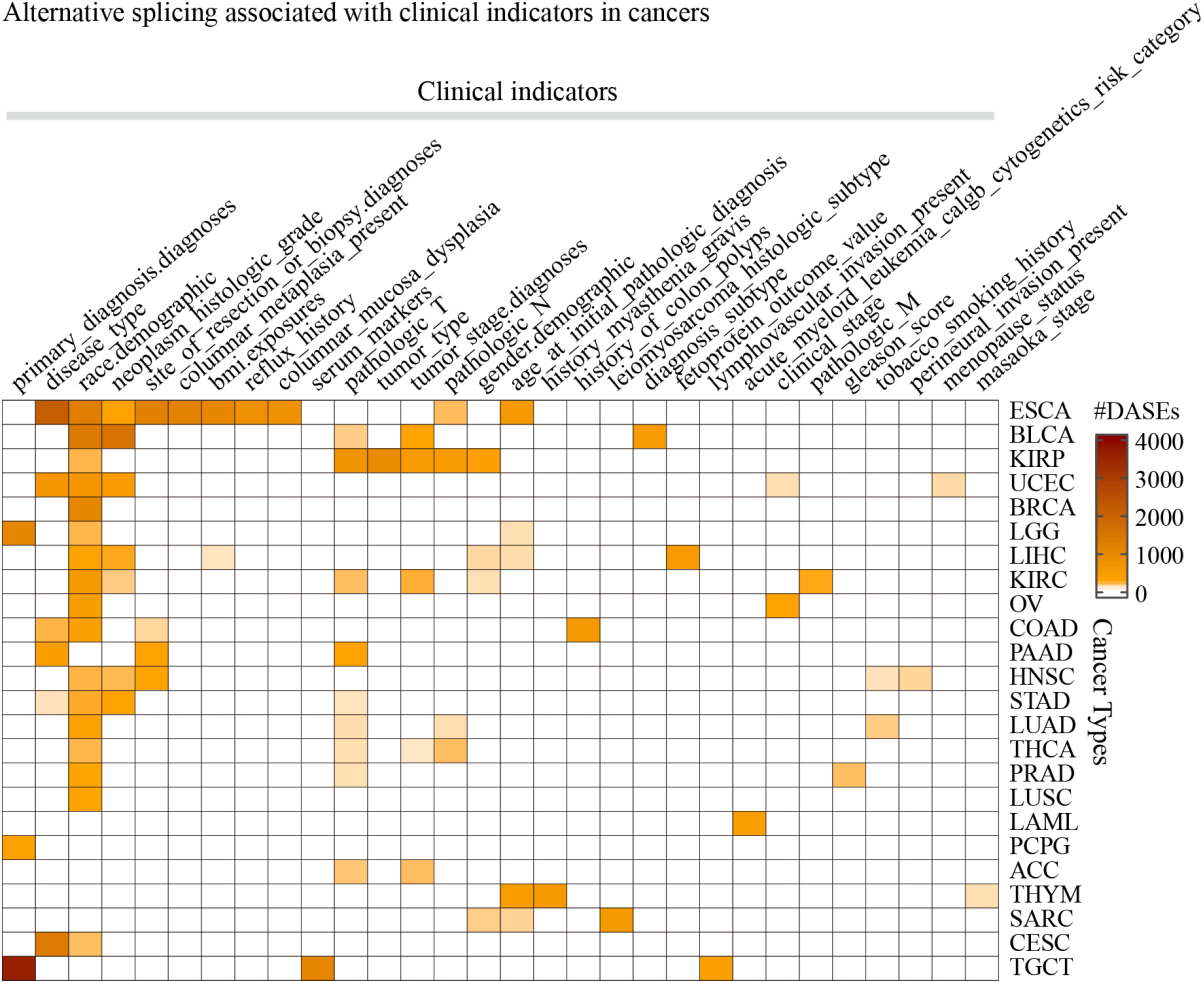
The statistics of the number of splicing events associated with clinical features in cancers. Only the 30 clinical features in 24 cancer types that were found associated with more than 50 ASEs were displayed.

Differential AS analysis was performed for each clinical feature in each cancer type for the splicing events whose non-null-PSI (Percent samples with values, PCT) was greater than 75%. The results showed that clinical features such as race (the number of cancers is 17), pathologic T (n = 9), neoplasm histologic grade (n = 7), and disease type (n = 6) were associated with at least 50 ASEs in more than 5 tumors (**Figure 2**; **Table S2**). On the other hand, ESCA has the most clinical features (the number of clinical features is 10) that were associated with AS, followed by KIRC, KIRP, and LIHC (n > 5). It is worth noting that there are also obvious differences in AS between different groups of age and gender in some cancer types, such as age in THYM and LIHC, and gender in KIRP and KIRC. The most significant splicing differences exist between different pathological subtypes or clinical diagnoses in the same cancer type, such as embryonal carcinoma and seminoma in TGCT (the number of ASEs is 3,593), adenocarcinomas and squamous cell carcinoma in ESCA (n = 2,097) or CESC (n = 1,344), adenocarcinoma and mucinous adenocarcinoma in UCEC (n = 603) or COAD (n = 166), papillary and non-papillary tumors in BLCA (n = 474). Further analysis showed that these clinical feature-related ASEs were mainly enriched in splice types AP, ES, and AT (**Figure S1**). These results indicate that significant differences in AS exist between different groups of many clinical features, such as different pathological subtypes, grades of the same tumor type as well as different races.

It is known that AS may influence immune cell infiltration in several facets. Here we further analyzed the immune cell infiltration in different groups of clinical features. We found that “B cell plasma” and “T cell CD4 memory resting” were significantly different between subgroups of primary diagnosis of LGG, and “T cell CD4 memory resting” and “T cell regulatory Tregs” were significantly different between subgroups of ESCA (**Figure S2**). Interestingly, there were a lot of ASEs identified between these subgroups of ESCA as well as LGG primary diagnosis (**Figure 2**). These results indicated that different strategies should be organized to deal with the patients in these different subgroups.

### Analysis of race-related AS

Race-related ASEs differ in most tumors (**Figure 2**). Al Abo et al. analyzed the differences in AS between African/Black Americans (BAs) and White Americans (WAs) in tumors using TCGA SpliceSeq data, but they did not compare the splicing differences between Asian Americans (AAs) and BAs and the roles of SNPs in producing race-related AS (29). Therefore, we further analyzed the differences in AS among different races in several tumors.

**Figure 3:**
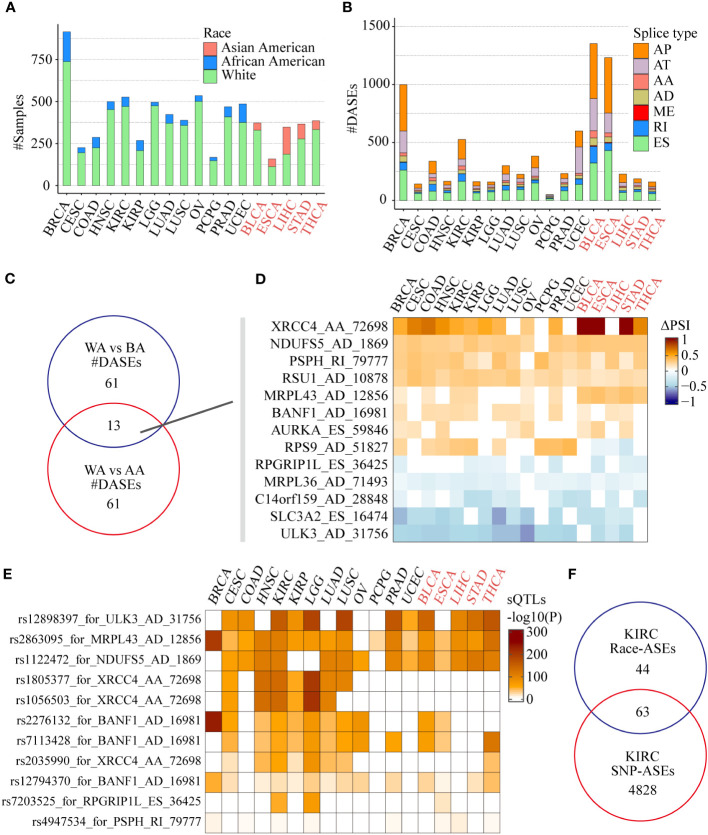
Race-related ASEs in the SpliceSeq project. **(A)** The number of samples of different races among the 18 tumors eligible for screening and differential analysis. **(B)** The number of splicing events in different splicing types associated with different races in each tumor. **(C)** Comparison of differential splicing events in the White and the Black, the White and the Asian American populations. **(D)** Splicing changes of the common ASEs of the 2 comparisons in each tumor. **(E)** Effects of SNPs on race-related splicing events and their differences in different tumors or races. **(F)** Comparison of 107 race-related ASEs (|ΔPSI| >0.1, FDR < 0.05) and 4891 SNP-related ASEs in the KIRC cohort (Fisher’s exact test P = 8.79e-10).

The results showed that tumors including BLCA, ESCA, LIHC, STAD, and THCA contain enough AAs for differential AS analysis compared with WAs, while other tumors such as BRCA and KIRC contain enough BAs for differential AS analysis compared with WAs (**Figure 3A**). We identified more than 1000 race-related ASEs in BRCA, BLCA, and ESCA, and more than 500 race-related ASEs in KIRC and UCEC, among which AP, AT, and ES accounted for the most (**Figure 3B**). To identify more significant race-related ASEs, considering the difference in the number of tumors involved, we required ASEs to be differential in at least 5 tumors when selecting race-related ASEs between WAs and BAs and selected ASEs between WAs and AAs requiring the presence of differences in at least 3 tumors. Finally, a total of 74 splicing events were identified as differential ASEs between WAs and BAs, another 74 splicing events were identified as significantly different between WAs and AAs, and there were 13 common splicing events in both comparisons (**Figure 3C**). Most of these common ASEs had the same directions of PSI changes in either comparison of WAs and BAs or comparison of WAs and AAs, except for RPS9_AD_51827 which was upregulated in WAs when compared with BAs while downregulated in WAs when compared with AAs (**Figure 3D**). Besides, race-related ASEs between WAs and BAs specifically included ARL6IP4_AA_25028, SLC25A26_ES_65549, PSMG4_RI_75176, and NMRAL1_AD_33740, while race-related ASEs between WAs and AAs specifically included ZDHHC4_ES_78754, TOR1AIP1_AA_9127, POMZP3_ES_80187, and IP6K2_RI_64750 (**Table S3**).

Among those 13 common race-related ASEs, there were 7 splicing events significantly correlated with nearby SNPs located between upstream and downstream 250 nt of the alternative exon. By using the search function of the CancerSplicingQTL database, it was found that splicing events such as ULK3_AD_31756, NDUFS5_AD_1869, and MRPL43_AD_12856 were significantly correlated with the corresponding SNPs rs12898397, rs2863095, and rs1122472 respectively in most of the analyzed cancers (**Figure 3E**). However, the relationship of significant correlations between XRCC4_AA_72698 and the corresponding SNPs rs1805377, rs1056503, and rs2035990 were only found in WAs and BAs tumors, which indicates that in addition to genomic factors, environmental factors such as lifestyle and diet may also play a role in tumors frequently occurring in Asian populations.

We further integrated race-related ASEs and sQTLs data in KIRC to comprehensively assess the impact of SNPs on ASEs. Considering the criteria of data filtering shown in the CancerSplicingQTL database: 1) splicing events exist in more than 90% of samples; 2) alternative exon is an independent single exon, the database included 16,225 ASEs (AA, AD, ES, and RI) in KIRC and 4891 ASEs were found significantly SNPs correlated. The data of race-related ASEs (|ΔPSI| >0.1, FDR < 0.05) in the KIRC cohort was screened further according to the above criteria, and 107 ASEs remained. After integrating with sQTLs data, we found that 63 (58.9%) of the race-related ASEs were SNP-significantly correlated ASEs (Fisher’s exact test P = 8.79e-10), including CAST_ES_72854, XRCC4_AA_72698 and Al Abo reported ASEs such as NEK3_ES_25994, ULK3_AD_31756, and NDUFS5_AD_1869 (**Figure 3F**). This data suggested that SNPs might be the main reason for the differences in alternative splicing in KIRC patients of different races.

### Clinical features-related AS in ESCA

**Figure 4:**
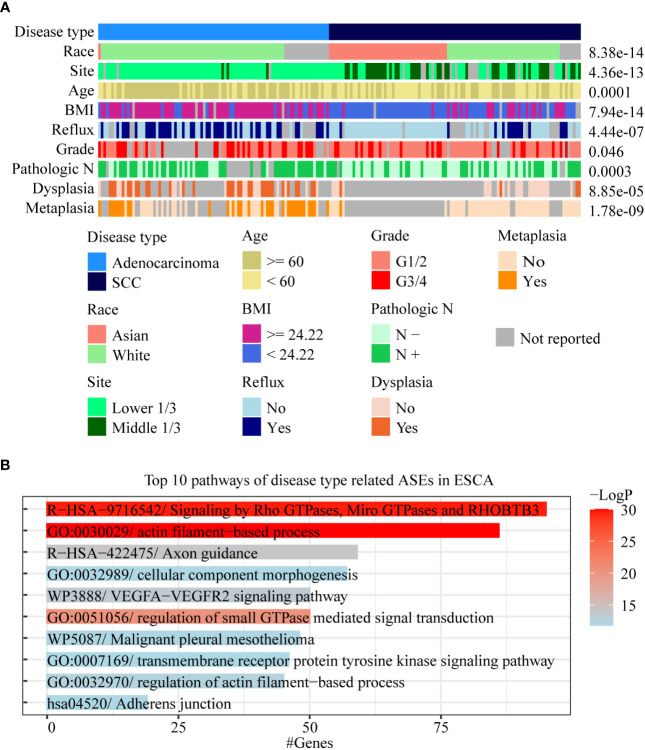
Analysis of AS-related clinical features in ESCA. **(A)** A waterfall plot was performed to display the relative relationships of AS-related clinical features, and the pathological disease type was set as the fundamental control. Fisher’s exact test was used to value the significance of the relationships between these clinical features with disease type. **(B)** Bar chart was used to show the results of gene function enrichment analysis of 1,018 genes within 1,717 disease type-related ASEs in the White Americans.

We found that there were multiple clinical features such as disease type, race, pathological grade, reflux history, and BMI, that were significantly correlated with ASEs in ESCA (**Figure 2**). Among these clinical features, the pathological disease type was found related to most ASEs, so the relationship between the disease type and other clinical features was investigated using a “waterfall” plot. In ESCA, pathological disease types were mainly divided into adenocarcinoma and squamous cell carcinoma. Fisher’s exact test showed that other clinical features were significantly correlated (overlapped) with pathological disease types, especially for race, tumor location, and BMI (P < 0.05, **Figure 4A**). Esophageal cancer in the Asian populations is mostly squamous cell carcinoma that occurs in the middle third of the esophagus, which may be related to an over-heat diet and other living habits (30). While in the White American population esophageal cancer is mostly adenocarcinoma that occurs in the lower third of the esophagus, which might be related to the incidence of gastroesophageal reflux followed by high BMI.

To effectively identify disease type- and race-related ASEs in ESCA, we further analyzed the splicing differences between adenocarcinoma and squamous cell carcinoma in WAs as well as splicing differences between AAs and WAs in squamous cell carcinoma. The results showed that 1,717 instead of 2,097 ASEs were identified after adjustment by race between different disease types and that 39 instead of 1,232 ASEs were identified after adjustment by disease type between different races. Gene function enrichment analysis showed that 1,018 genes within these 1,717 disease-type-related ASEs were mainly enriched to pathways such as actin filament-based process and signaling by Rho GTPases (**Figure 4B**). Among these 39 race-related differential ASEs, 23 were identified as race-related between WAs and BAs in at least 5 cancer types. These results indicated that disease type might decide the splicing differences in ESCA, and that race might only contribute a few to these differences. Moreover, race-related ASEs can be found more effectively by requiring of existing in several cancer types at the same time.

### Function enrichment analysis of clinical feature-related ASEs in cancers

**Figure 5:**
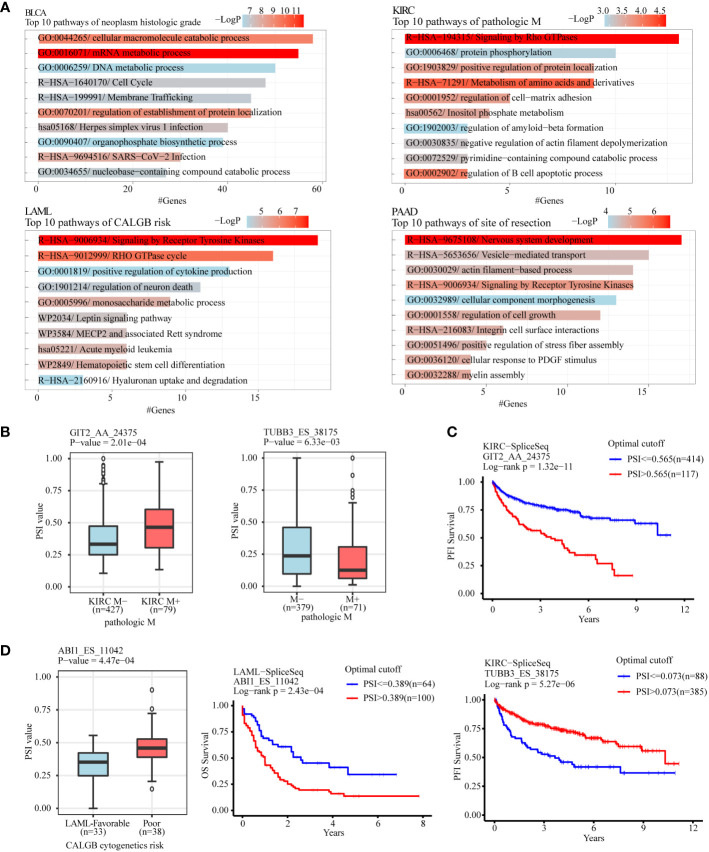
Gene function enrichment analysis of clinical feature-related ASEs in cancers. **(A)** Clinical feature-related ASEs in cancers including “neoplasm histologic grade” in BLCA, “pathologic M” in KIRC, “acute myeloid leukemia calgb cytogenetics risk category” in LAML, and “site of resection” in PAAD were selected to perform gene function enrichment analysis. **(B)** GIT2 and TUBB3 were selected as 2 examples of splicing genes belonging to the enriched pathway “Signaling by Rho GTPases” of pathologic metastasis-related ASEs in the KIRC cohort. **(C)** Kaplan-Meier plot showed that AS of GIT2 and TUBB3 was both significantly associated with KIRC patients’ progression-free survival. **(D)** ABI1 was selected as an example of splicing genes belonging to the enriched pathway “Signaling by Receptor Tyrosine Kinases” of CALGB cytogenetics risk-related ASEs in the LAML cohort. AS of ABI1 was significantly associated with LAML patients’ overall survival.

To analyze the gene function of ASEs related to different clinical features in tumors, clinical features with considerable ASEs in cancers were selected to perform gene function enrichment analysis, including “neoplasm histologic grade” in BLCA, “pathologic M” in KIRC, “acute myeloid leukemia calgb cytogenetics risk category” in LAML, “site of resection” in PAAD, “history of colon polyps” in COAD, “fetoprotein outcome value” in LIHC, “tobacco smoking history” in LUAD, and “gender” in KIRP (**Figures 5A**; **S3A**). The results showed that the “GO:0016071/mRNA metabolic process” and “GO:0044265/cellular macromolecule catabolic process” were the most 2 significant enrichment pathways of ASEs related to “neoplasm histologic grade” in BLCA. “R−HSA−194315/Signaling by Rho GTPases” and “R−HSA−71291/Metabolism of amino acids and derivatives” were the most 2 pathways of “pathologic M” in KIRC. “R−HSA−9675108/Nervous system development” and “R−HSA−9006934/Signaling by Receptor Tyrosine Kinases” were the most 2 pathways of “site of resection”-related ASEs in PAAD. “R−HSA−9006934/Signaling by Receptor Tyrosine Kinases” and “R−HSA−9012999/RHO GTPase cycle” were the most 2 pathways of “CALGB cytogenetics risk”-related ASEs in LAML (**Figure 5A**). “GO:0032878/regulation of establishment of cell polarity” and “GO:0000278/mitotic cell cycle” were the most 2 pathways of the “history of colon polyps” in COAD. “WP2882/Nuclear receptors meta−pathway” and “R−HSA−382551/Transport of small molecules” were the 2 pathways of “fetoprotein outcome value” in LIHC. “hsa05168/Herpes simplex virus 1 infection” and “R−HSA−382551/Transport of small molecules” were the most two pathways of “gender” in KIRP. “WP3678/Amplification and expansion of metastatic traits” and “GO:0006664/glycolipid metabolic process” were the most 2 pathways of “tobacco smoking history” in LUAD (**Figure S3A**).

For example, the pathway “Signaling by Rho GTPases” showed a significant association with pathological metastasis of KIRC. Among genes in this pathway, AS of GIT2 (GIT2_AA_24375), showed a significant increase in metastasis tissues compared with no-metastasis tissues and was negatively associated with progression-free survival of KIRC patients, while AS of TUBB3 (TUBB3_ES_38175) showed a significant decrease in metastasis tissues and was positively associated with progression-free survival of KIRC patients (**Figures 5B, C**). The pathway “Signaling by Receptor Tyrosine Kinases” showed a significant association with the “CALGB cytogenetics risk” of LAML. AS of ABI2 (ABI1_ES_11042), a key gene in this pathway, showed a significant increase in the poor category compared with the favorable category and was associated with poor overall survival of LAML patients (**Figure 5D**). The pathway “regulation of establishment or maintenance of cell polarity” also significantly enriched the “history of colon polyps”-related ASEs in COAD. Among genes in this pathway, AS of LLGL2 (LLGL2_AP_43458) showed a significant increase in COAD with a history of colon polyps when compared to COAD patients without (**Figure S3B**). The pathway “Nuclear receptors meta-pathway” significantly enriched with the “fetoprotein outcome value”-related ASEs in LIHC. Among these, AS of SCP2 (SCP2_ES_3045) and SLC27A5 (SLC27A5_AP_52472) showed a significant difference in LIHC with different fetoprotein levels and were both. associated with LIHC patients’ overall survival (**Figure S3C, D**).

### Identification of RBPs to regulate clinical feature-related ASEs

**Figure 6:**
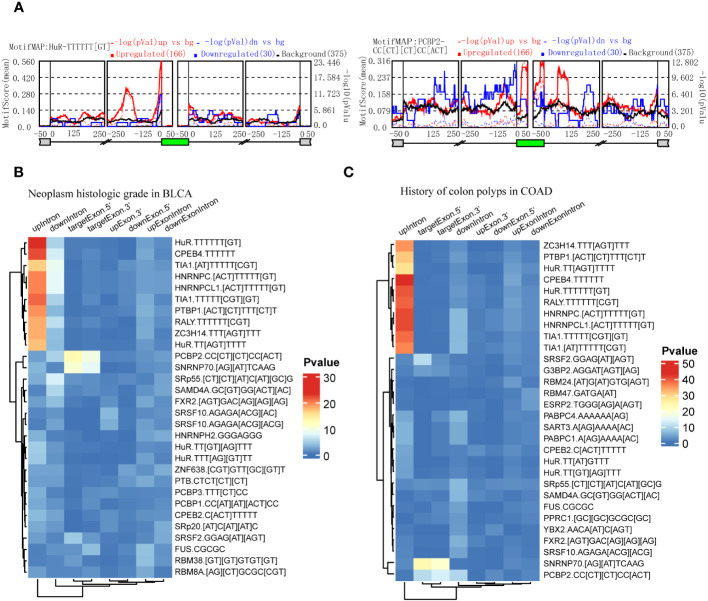
Identification of RBPs contributing to the regulation of clinical feature-related ASEs by using rMAPS2. **(A)** HuR and PCBP2 were selected as the 2 most representative RBPs that were enriched to Exon Skipping splicing events in neoplasm histologic grade-related ASEs in BLCA. **(B)** Heatmap showed the most 30 significant enrichments of RBP-motifs to the neoplasm histologic grade-related ASEs in BLCA. **(C)** Heatmap showed the most 30 significant enrichments of RBP-motifs to the colon polyps-related ASEs in COAD.

RBPs could be predicted by the enrichment of motifs around alternative exon for ASEs in splice types AA, AD, ES, RI, and ME respectively. We identified clinical feature-related ASEs in several cancer types (**Figure 2**). However, these clinical feature-related ASEs were in different proportions of splice types, and exon skipping (ES) accounted for the most (**Figure S1**). To explore the potential regulators of “neoplasm histologic grade”-related ASEs in BLCA and “history of colon polyps”-related ASEs in COAD, exon skipping (ES) events were extracted with genome coordination and organized into the format that meets the requirements of rMAPS2, due to the considerable amounts of AS events. The results showed that ASEs up-regulated in BLCA with high-level neoplasm grade were enriched to several RBPs’ motifs, including “poly-T” motifs and polypyrimidine tract (Py-tract) sequence of HuR, CPEB4, TIA1, HNRNPC, PTBP1, RALY and ZC3H14, and motifs of PCBP2 and SNRNP70 (**Figures 6A, B**). ASEs upregulated in COAD without a history of colon polyps were similarly enriched to RBP motifs of neoplasm grade-related ASEs in BLCA (**Figure 6C**). Py-tract sequence was often located in the upstream intron of alternative exon and used to attract spliceosome through the recognition of RBPs to the 3’ splice site, resulting in the inclusion of alternative exons (31). The inclusion of alternative exons of these Py-tract ASEs indicated that mechanisms suppressing exon inclusion were wiped out in BLCA with high-grade pathology. In addition, PCBP2 and SNRNP70 might also play important roles in affecting AS regulation in these different clinical feature groups.

### Validation of clinical feature-related ASEs and predicted RBPs

**Figure 7:**
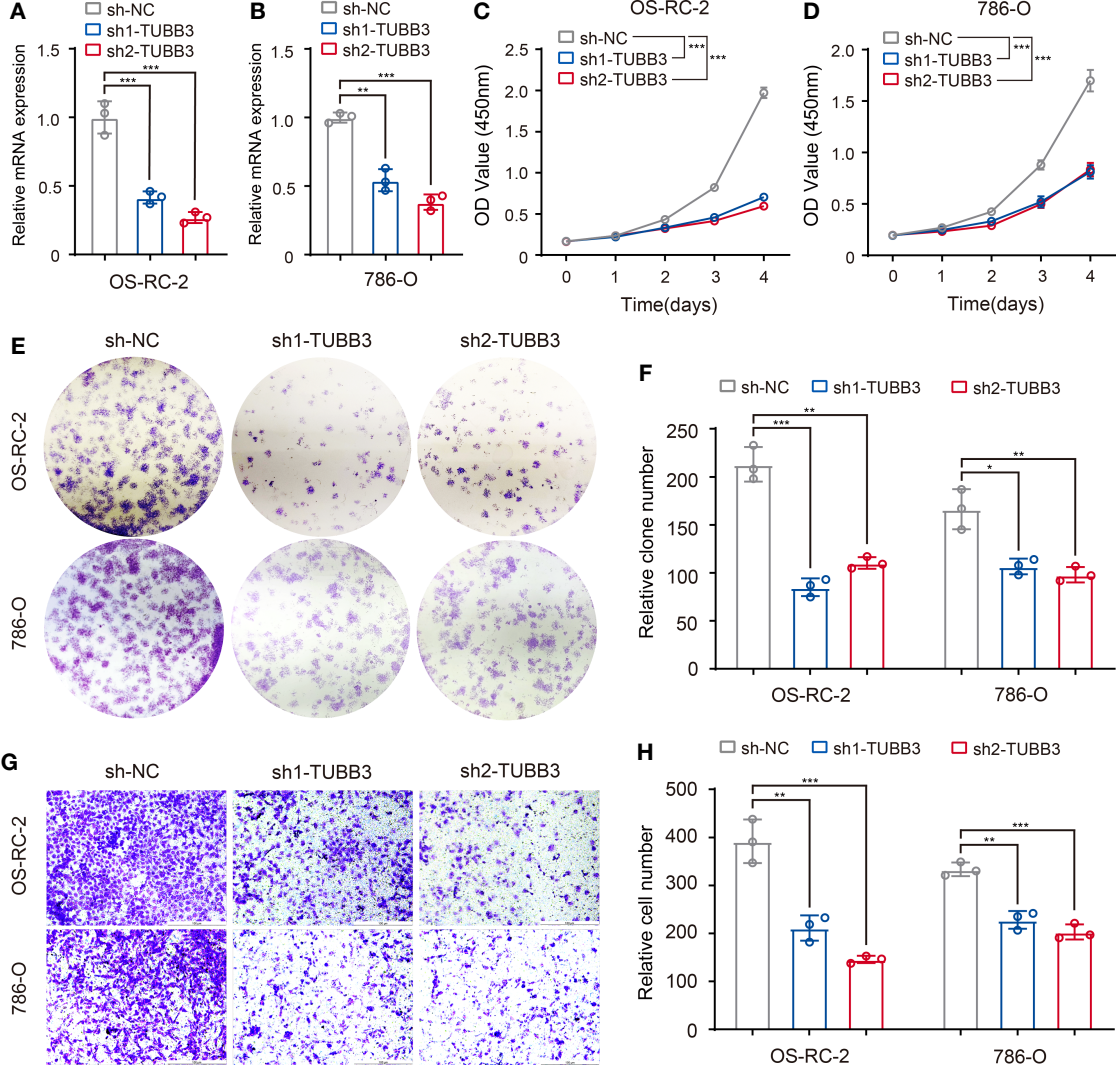
Validation of metastasis-related ASEs (TUBB3_ES_38175) in kidney cancer cells. **(A, B)** Expression of TUBB3 mRNA was determined by qPCR in OS-RC-2 and 786-O cells with TUBB3 knockdown. **(C, D)** CCK-8 assays were performed in OS-RC-2 and 786-O cells after sh1-TUBB3, sh2-TUBB3 and sh-NC transfection. **(E, F)** Colony formation assays were performed in OS-RC-2 and 786-O cells after sh1-TUBB3, sh2-TUBB3 and sh-NC transfection. **(G, H)** Modified Boyden chamber assays were applied for migration of OS-RC-2 and 786-O cells after sh1-TUBB3, sh2-TUBB3 and sh-NC transfection. The data are shown as the mean ± SD. One representative plot of n = 3 experiments is shown. *, P < 0.05; **, P < 0.01, ***, P < 0.001, as determined by one-way **(A, B, F, H)** or two-way **(C, D)** ANOVA.

To validate the gene function of ASEs related to different clinical features, we chose a metastasis-related ASE in KIRC (TUBB3_ES_38175) as analyzed in **Figure 5**, and performed experiments to demonstrate the relationship between AS of TUBB3 and metastatic potency of kidney cancer cells. As TUBB3_ES_38175 caused TUBB3 mRNA degradation, we transfected TUBB3 shRNA in OS-RC-2 and 786-O cell lines and verified decreased TUBB3 expression (**Figures 7A, B**). CCK-8 (**Figures 7C, D**) and clone formation assays (**Figures 7E, F**) showed that TUBB3 silencing decreased the proliferation and clonogenic ability in OS-RC-2 and 786-O cells. Importantly, we demonstrated that TUBB3 knock-down significantly weakened the migration ability of OS-RC-2 and 786-O cells (**Figures 7G, H**), which was consistent with our function enrichment analysis of pathological metastasis-related ASEs in KIRC.

**Figure 8:**
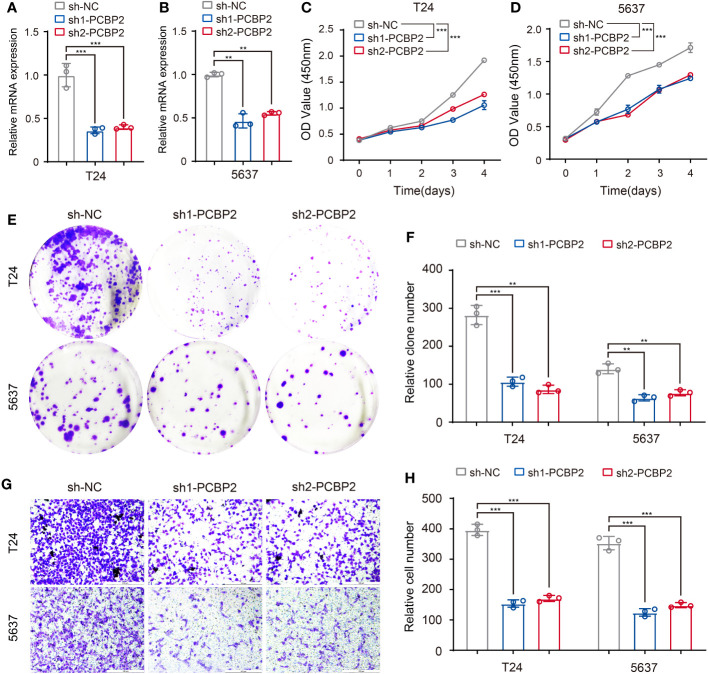
Validation of identified RBP (PCBP2) in tumor cells. **(A, B)** Expression of PCBP2 mRNA was determined by qPCR in T24 and 5637 cells with PCBP2 knocked down. **(C, D)** CCK-8 assays were performed in T24 and 5637 cells after sh1-PCBP2, sh2-PCBP2 and sh-NC transfection. **(E, F)** Cell colony formation assays were performed in T24 and 5637 cells after sh1-PCBP2, sh2-PCBP2 and sh-NC transfection. **(G, H)** Transwell assays were applied for migration of T24 and 5637 cells after sh1-PCBP2, sh2-PCBP2 and sh-NC transfection. The data are shown as the mean ± SD. One representative plot of n = 3 experiments is shown. **P < 0.01, ***P < 0.001, as determined by one-way **(A, B, F, H)** or two-way **(C, D)** ANOVA.

To preliminarily validate the relationship between the above-identified RBPs and associated clinicopathologic features, we explored the role of PCBP2 in the proliferative and migratory ability of bladder cancer cells given the potential regulation of PCBP2 to “neoplasm histologic grade”-related ASEs in BLCA. Human bladder cancer cell lines (T24 and 5637) with PCBP2 stably knocked down were constructed and validated (**Figures 8A, B**). CCK-8 (**Figures 8C, D**) and clone formation assays (**Figures 8E, F**) showed that PCBP2 silencing decreased the proliferation and clonogenic ability in T24 and 5637 cells. Furthermore, we observed that the migration ability was weakened in PCBP2 knock-down T24 and 5637 cells (**Figures 8G, H**). These results suggested that PCBP2 promoted the malignancy of bladder cancer cells, which was related to the high-grade pathology of BLCA.

## Discussion

In this study, we systematically analyzed AS differences between different groups of 58 clinical features in 33 cancers, and identified 30 clinical features in 24 cancer types associated with more than 50 ASEs individually. Among these, we found that pathological subtypes might represent the main resource of splicing differences in a cancer type. Race-related ASEs may result from SNPs that vary in different races. Gene function enrichment analyses showed that “pathologic M”-related ASEs in KIRC mainly enriched to “Signaling by Rho GTPases” pathway, “neoplasm histologic grade”-related ASEs in BLCA mainly enriched to “mRNA metabolic process”, “CALGB cytogenetics risk category”-related ASEs in LAML mainly enriched to “Signaling by Receptor Tyrosine Kinases”. Among those ASEs associated with metastasis, AS of GIT2 and TUBB3 might be 2 represents in KIRC metastasis. While among those ASEs associated with CALGB cytogenetics risk, AS of ABI1 might be an effective representation in LAML. Finally, we identified several RBPs such as PCBP2, SNRNP70, HuR, and TIA1, which might contribute to splicing differences between different groups of neoplasm grade in BLCA. Our work systematically revealed the relationship between AS and clinicopathological features in cancers, providing novel insights for tumor progression and promising molecular targets for cancer therapy by regulating clinically relevant AS and splicing factors.

As aberrant AS has been discovered to be a vital contributor to tumorigenesis and cause of tumor heterogeneity, growing evidence reveals the clinical implications of specific aberrant ASEs in cancers. Among these, most reports focus on the relationship between aberrant ASEs and prognosis and survival in cancer patients. After identification of the overall survival-associated ASEs, prognostic models were developed to predict the survival outcomes of patients with aberrant AS patterns in gastric cancer (32–35), breast cancer (36), CHOL (37), glioblastoma (38), pancreatic cancer (39), LAML (40), UVM (41), HNSC (42), etc. Aberrant AS is also related to chemotherapy resistance. Androgen-receptor splice variant (AR-V7), a transcript generated by the AS mechanism, is responsible for the resistance to enzalutamide and abiraterone in castration-resistant prostate cancer (43). In addition, there are a few studies that reported the relationship between AS and pathological characteristics of tumors. Belluti et al. suggested that different NF-YA isoforms led to different phenotypes of prostate cancers (44). In oral squamous cell carcinoma, TGIF1 splicing variant 8 was reported to be correlated with the pathologic stage (45). In our previous study, we characterized large amounts of different ASEs in 2 pathological subtypes of testicular germ cell tumors (46). However, ASEs related to diverse clinicopathologic characteristics are still rarely systematically profiled in pan-cancers. Herein, the present study included 58 clinicopathologic characteristics of cancers in differential AS analysis and identified clinically relevant ASEs across multiple cancers. A considerable number of ASEs were found to be associated with clinical features such as race, pathologic T, neoplasm histologic grade and disease type. And profiles of clinically relevant ASEs varied with tumor types. For example, in KIRC, different ASEs were mainly associated with pathological metastasis, and function enrichment analysis showed that these ASEs were enriched in pathway “Signaling by Rho GTPases”, which was considered a critical pathway involved in cancer metastasis (47).

Although the relationship between aberrant ASEs and tumor heterogeneity is well characterized, how ASEs contribute to tumor heterogeneity and affect tumorigenesis and tumor progression remains unclear. It is generally accepted that aberrant AS affects the expression patterns of tumor-related genes. Mechanically, aberrant AS may create a premature termination codon (PTC) which can be recognized by the intracellular mRNA quality surveillance system and then trigger the activation of the nonsense-mediated mRNA decay (NMD) pathway (48). For instance, Wollerton et al. revealed that AS of polypyrimidine tract binding protein (PTB) created mRNA isoforms that were destructed during NMD (49). Interestingly, we found that the ASE “TUBB3_ES_38175” was associated with pathological metastasis in KIRC. However, the ASE “TUBB3_ES_38175” generated a TUBB3 mRNA isoform that could be removed by NMD, and was down-regulated in metastatic cancer. This AS event, in turn, increased the expression of TUBB3, which was evidenced to promote the progression of KIRC and also validated by our experiments *in vitro* (50). The ASE “TUBB3_ES_38175” could serve as a marker of tumor heterogeneity in KIRC to evaluate tumor metastasis, guide cancer therapies, and deepen our understanding of the mechanism of cancer progression. Therefore, our result indicated that the occurrence of specific ASEs could exert anti-tumor effects.

Given that AS plays such a critical role in the genesis and development of multiple cancer types, it is urgent to clarify the modulation of AS. As a nuclear process mediated by spliceosome formed by small nuclear ribonucleoproteins (snRNPs), AS is regulated mainly by cis-acting elements and trans-acting RBPs including splicing factors. Through whole-exome and RNA sequencing, the somatic mutation in splicing regulatory cis-elements was shown to affect AS in cancers (51). In the present study, we revealed the relationship between ASEs and nearby SNPs in different races, suggesting that mutations in cis-elements decided race-related ASEs across cancer types. In addition, numerous studies reported that mutations and epigenetic regulation of trans-factors accounted for aberrant ASEs in cancers. Frequent somatic mutations in splicing factors including SF3B1, SRSF2, and U2AF1 were discovered in myeloid malignancies as well as solid tumors (52–59). The expression of splicing factors was also reported to be regulated by DNA methylation and histone modification (60–62). Furthermore, a transcription factors-RBPs-AS triplet analysis was used to interpret aberrant ASEs in cancer (63). Our results identified RBPs including PCBP2 and SNRNP70 responsible for differential ASEs upregulated in BLCA with high-level neoplasm grade. However, the functions and modulations of identified RBPs in cancers need further investigation in experimental and clinical studies.

To sum up, through detailed analysis of the differences in AS among the clinical features of tumors, we found that there are significant correlations between multiple clinical features such as race, disease type, age and gender, and AS. These differences may be affected by SNPs or splicing factors. Although we see associations between clinical features, which somewhat downplays differences in AS among other clinical features, however, this splicing difference plays a role in the differential development of the disease. What kind of effect is not yet achieved, and still needs a lot of further research.

## Data availability statement

The original contributions presented in the study are included in the article/**Supplementary Material**. Further inquiries can be directed to the corresponding authors.

## Author contributions

Conceptualization, CD, YZ, ZH, and HX; methodology, validation, CD and YZ; data collection and analyzing, LL, KL, XY, SL, and YG; writing, CD, YZ, YG, and HX; data curation, XW, BL, XM, and HW; project administration, JZ, HL, ZH, and HX. All authors contributed to the article and approved the submitted version.

## Supplementary material

Supplementary Figure 1 The statistics of the number of splicing events in different splicing types associated with clinical features in cancers. Only the 30 clinical features in 24 cancer types that were associated with more than 50 ASEs were displayed.

Supplementary Figure 2 The statistics of the significance of differences in immune cell infiltration between 2 groups of clinical features in cancers. TCGA-CIBERSORT project data was used to perform differential analysis. Only the 14 immune cells and 23 clinical features in 21 cancer types with a delta proportion of more than 3 percent and BH adjust P value of less than 0.05 were kept and displayed.

Supplementary Figure 3 Gene function enrichment analysis of clinical feature-related ASEs in cancers. **(A)** Clinical feature-related ASEs in cancers including “history of colon polyps” in COAD, “fetoprotein outcome value” in LIHC, “tobacco smoking history” in LUAD, and “gender” in KIRP were selected to perform gene function enrichment analysis. **(B)** LLGL2 and ECT2 were selected as an example of splicing genes respectively belonging to the enriched pathways “regulation of establishment or maintenance of cell polarity” and “mitotic cell cycle” of colon polyps-related ASEs in the COAD cohort. **(C)** SLC27A5 and SCP2 were selected as 2 examples of splicing genes belonging to the enriched pathway “Nuclear receptors meta-pathway” of fetoprotein outcome value-related ASEs in the LIHC cohort. **(D)** Kaplan-Meier plots showed that AS of SLC27A5 and SCP2 was significantly associated with LIHC patients’ overall survival.
