# Supplementary figures and images for: Identification of alternative splicing associated with clinical features: from pan-cancers to genitourinary tumors
Source: Front Oncol. 2023 Sep 25;13:1249932. doi: 10.3389/fonc.2023.1249932 (PMC10557043; doi:10.3389/fonc.2023.1249932)

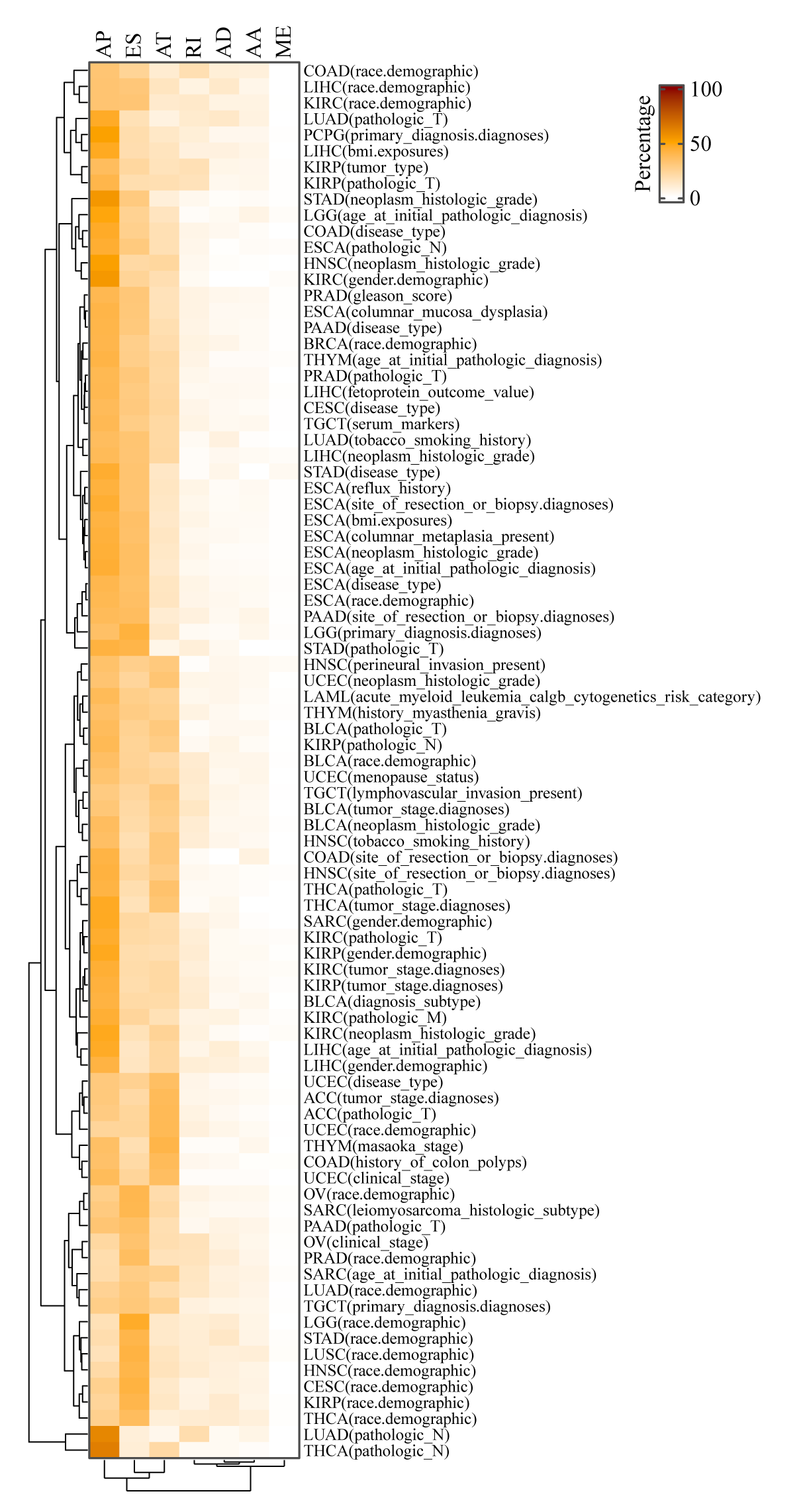

Supplement: Supplementary Figure 1 — The statistics of the number of splicing events in different splicing types associated with clinical features in cancers. Only the 30 clinical features in 24 cancer types that were associated with more than 50 ASEs were displayed. [file Image_1.tif]

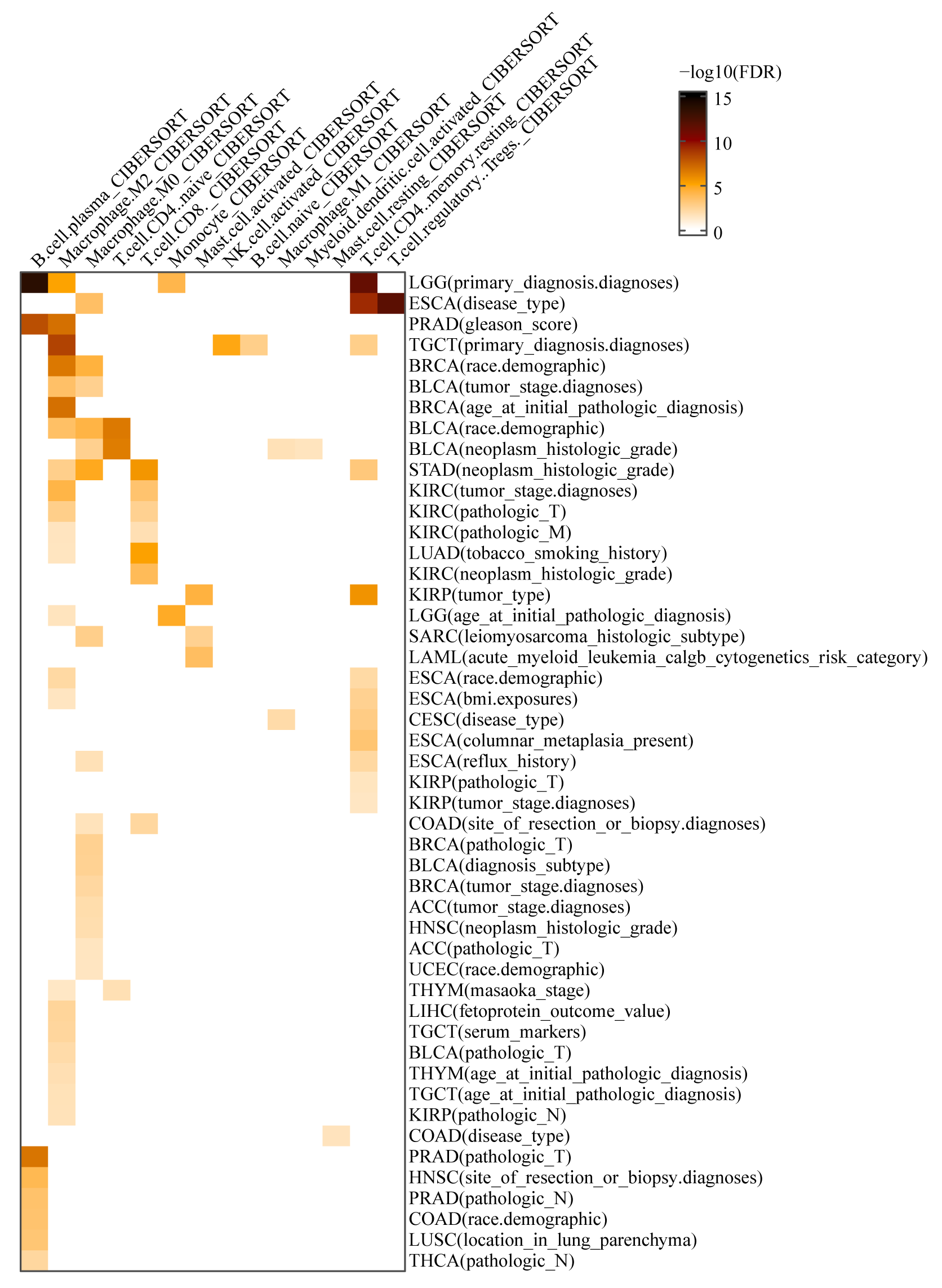

Supplement: Supplementary Figure 2 — The statistics of the significance of differences in immune cell infiltration between 2 groups of clinical features in cancers. TCGA-CIBERSORT project data was used to perform differential analysis. Only the 14 immune cells and 23 clinical features in 21 cancer types with a delta proportion of more than 3 percent and BH adjust P value of less than 0.05 were kept and displayed. [file Image_2.tif]

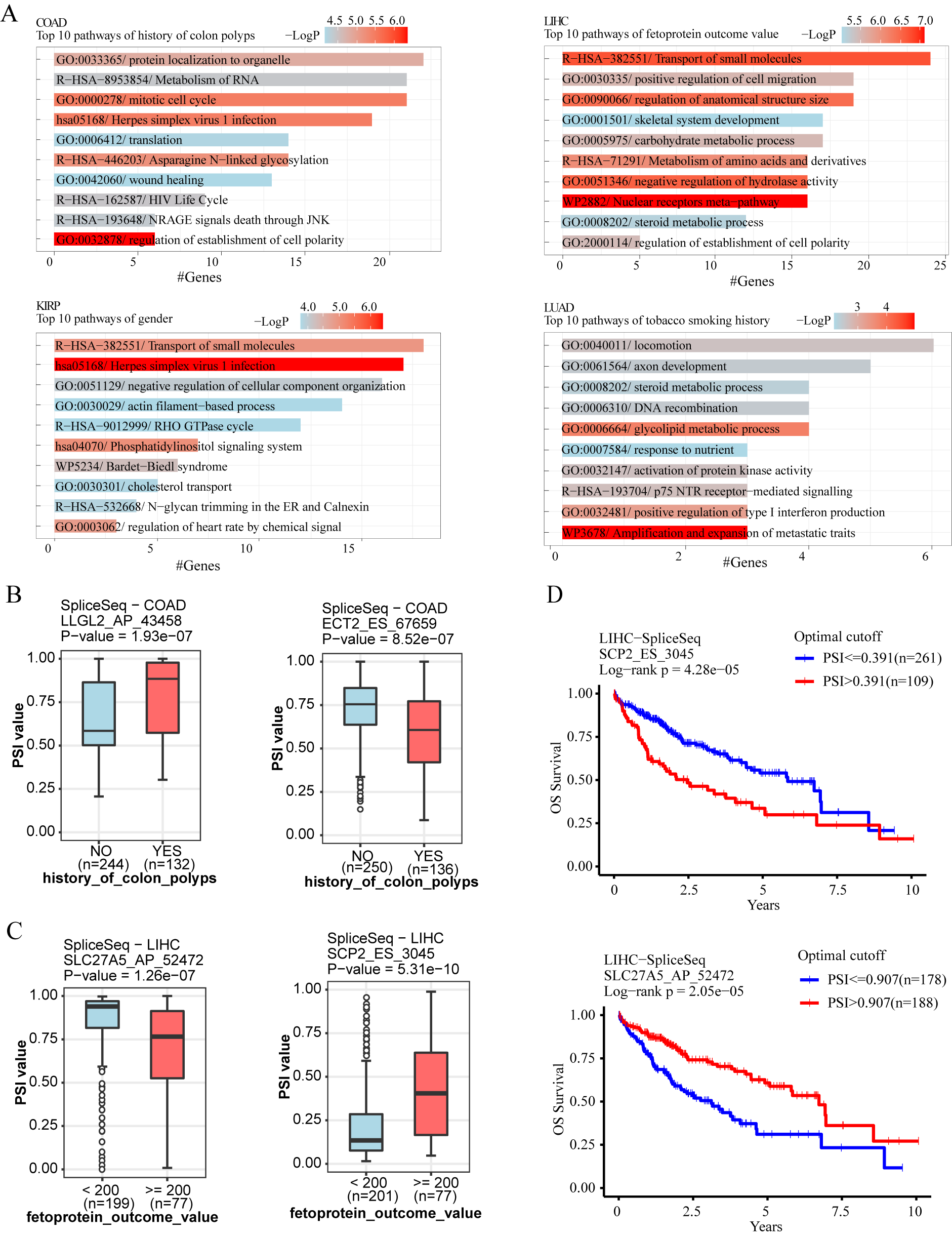

Supplement: Supplementary Figure 3 — Gene function enrichment analysis of clinical feature-related ASEs in cancers. (A) Clinical feature-related ASEs in cancers including “history of colon polyps” in COAD, “fetoprotein outcome value” in LIHC, “tobacco smoking history” in LUAD, and “gender” in KIRP were selected to perform gene function enrichment analysis. (B) LLGL2 and ECT2 were selected as an example of splicing genes respectively belonging to the enriched pathways “regulation of establishment or maintenance of cell polarity” and “mitotic cell cycle” of colon polyps-related ASEs in the COAD cohort. (C) SLC27A5 and SCP2 were selected as 2 examples of splicing genes belonging to the enriched pathway “Nuclear receptors meta-pathway” of fetoprotein outcome value-related ASEs in the LIHC cohort. (D) Kaplan-Meier plots showed that AS of SLC27A5 and SCP2 was significantly associated with LIHC patients’ overall survival. [file Image_3.tif]

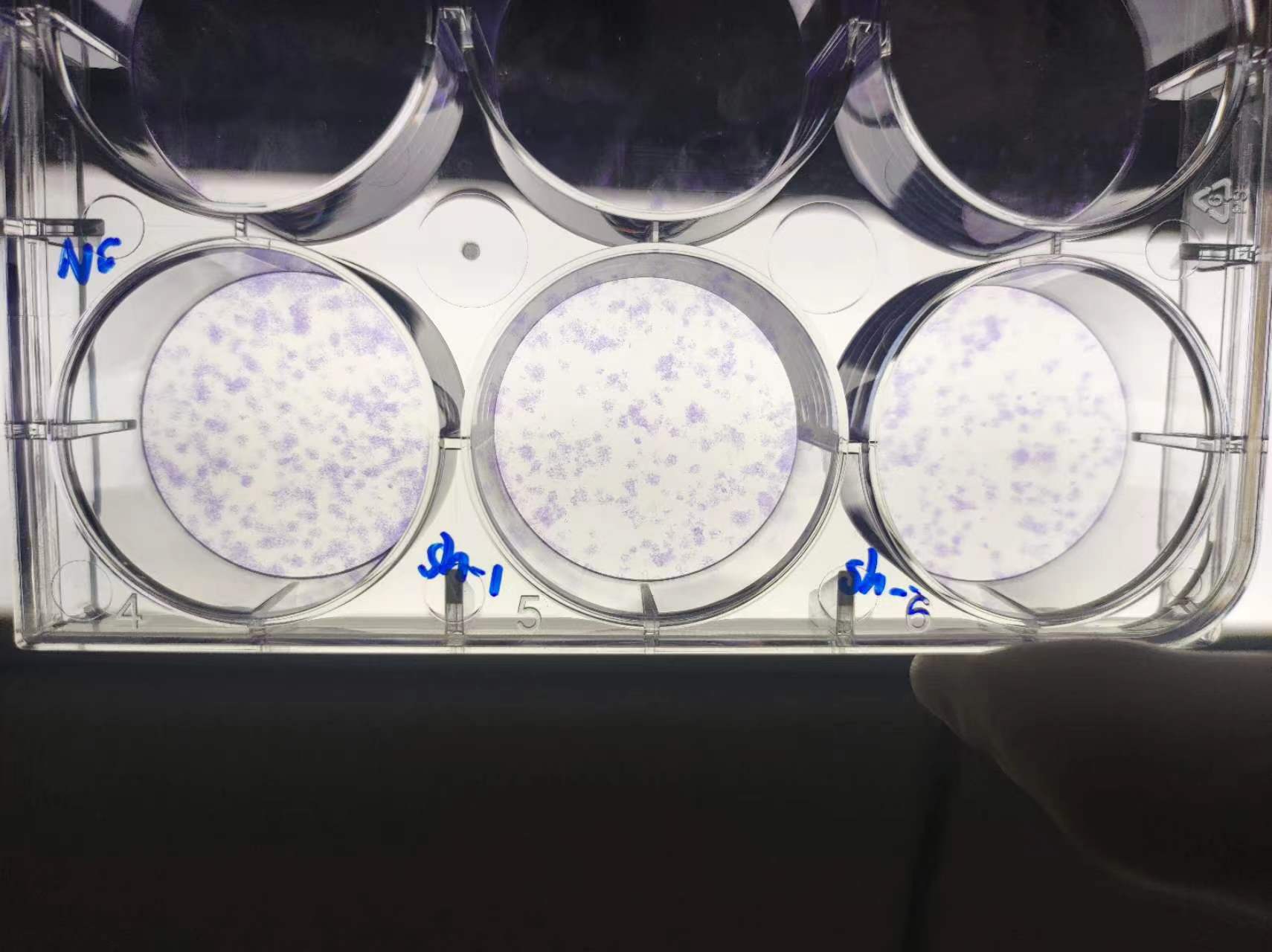

Supplement: Supplementary file 7 [file DataSheet_1.zip › Raw Data/microscopy images/Figure7E/786-O-clones.jpg]

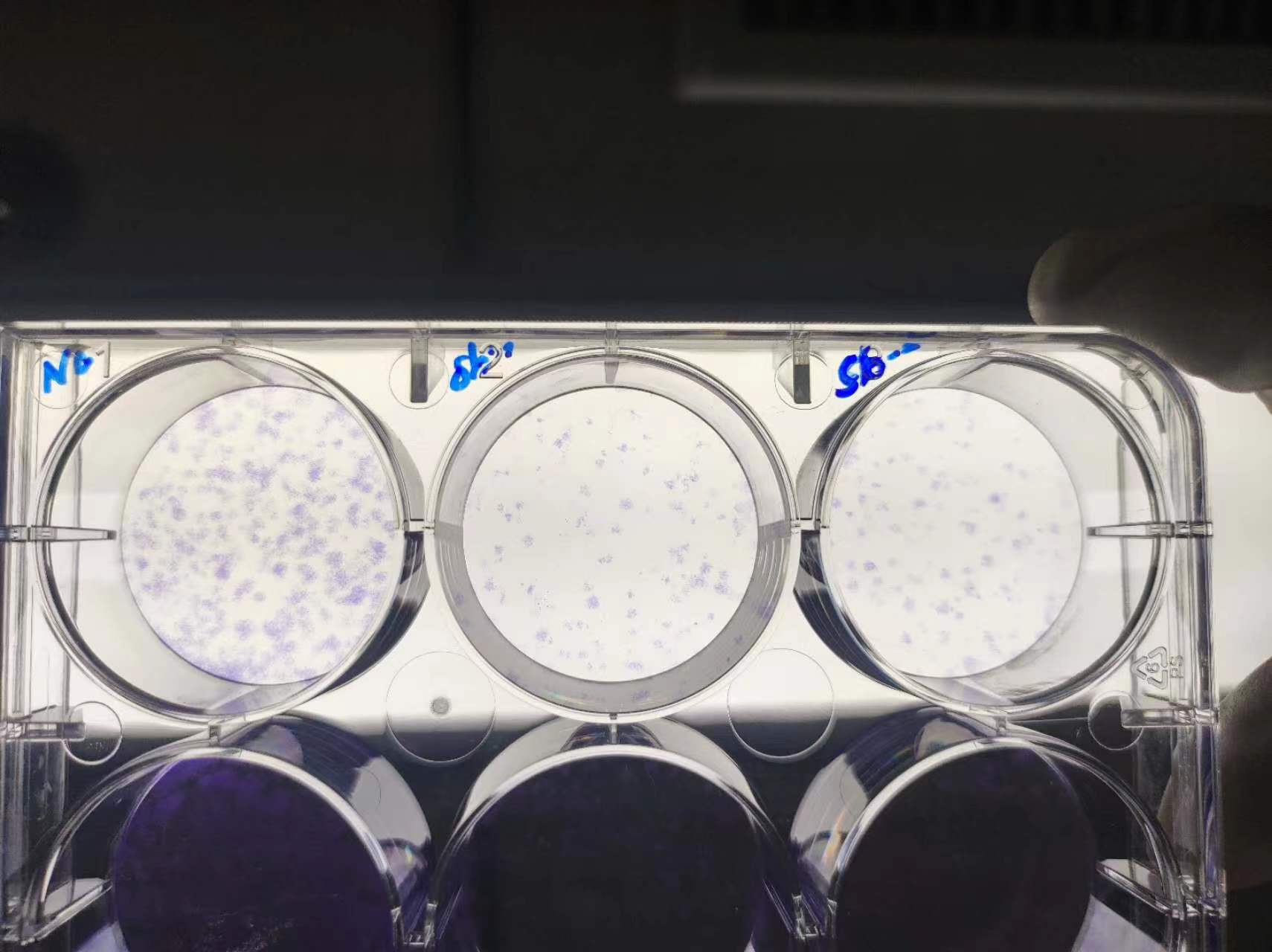

Supplement: Supplementary file 7 [file DataSheet_1.zip › Raw Data/microscopy images/Figure7E/OS-clones.jpg]

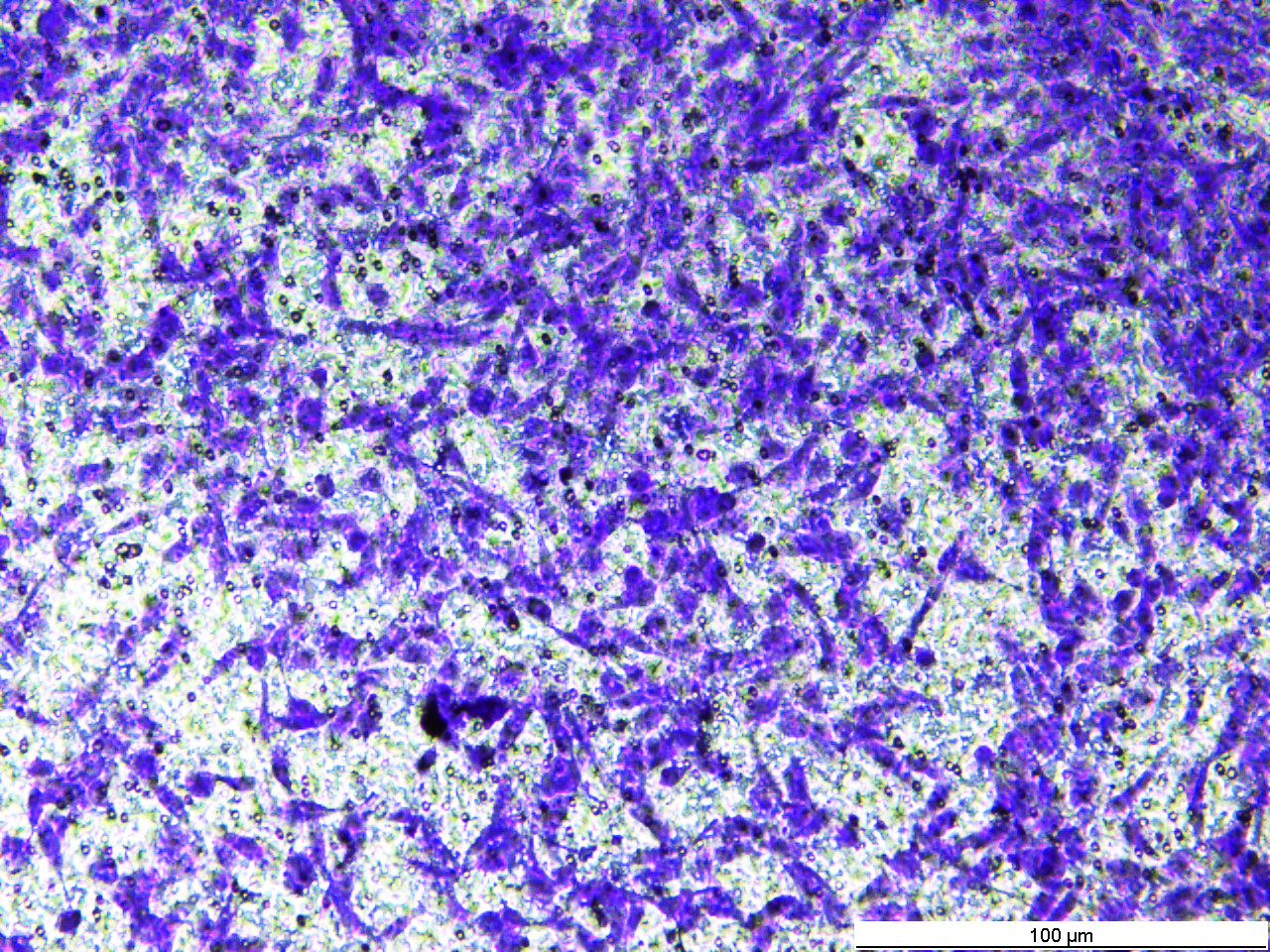

Supplement: Supplementary file 7 [file DataSheet_1.zip › Raw Data/microscopy images/Figure7G/Figure7G 786O-NC.jpg]

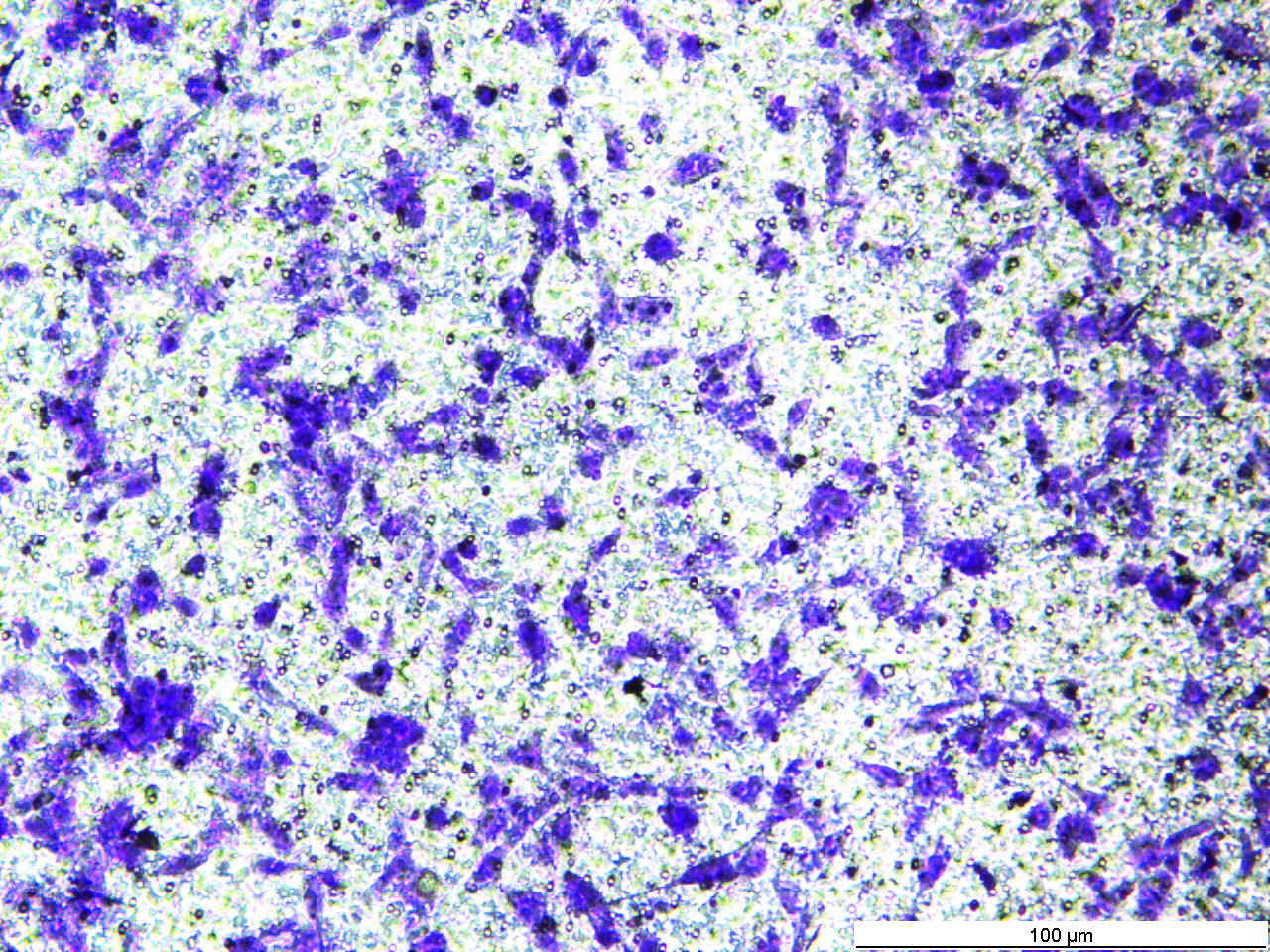

Supplement: Supplementary file 7 [file DataSheet_1.zip › Raw Data/microscopy images/Figure7G/Figure7G 786O-sh1.jpg]

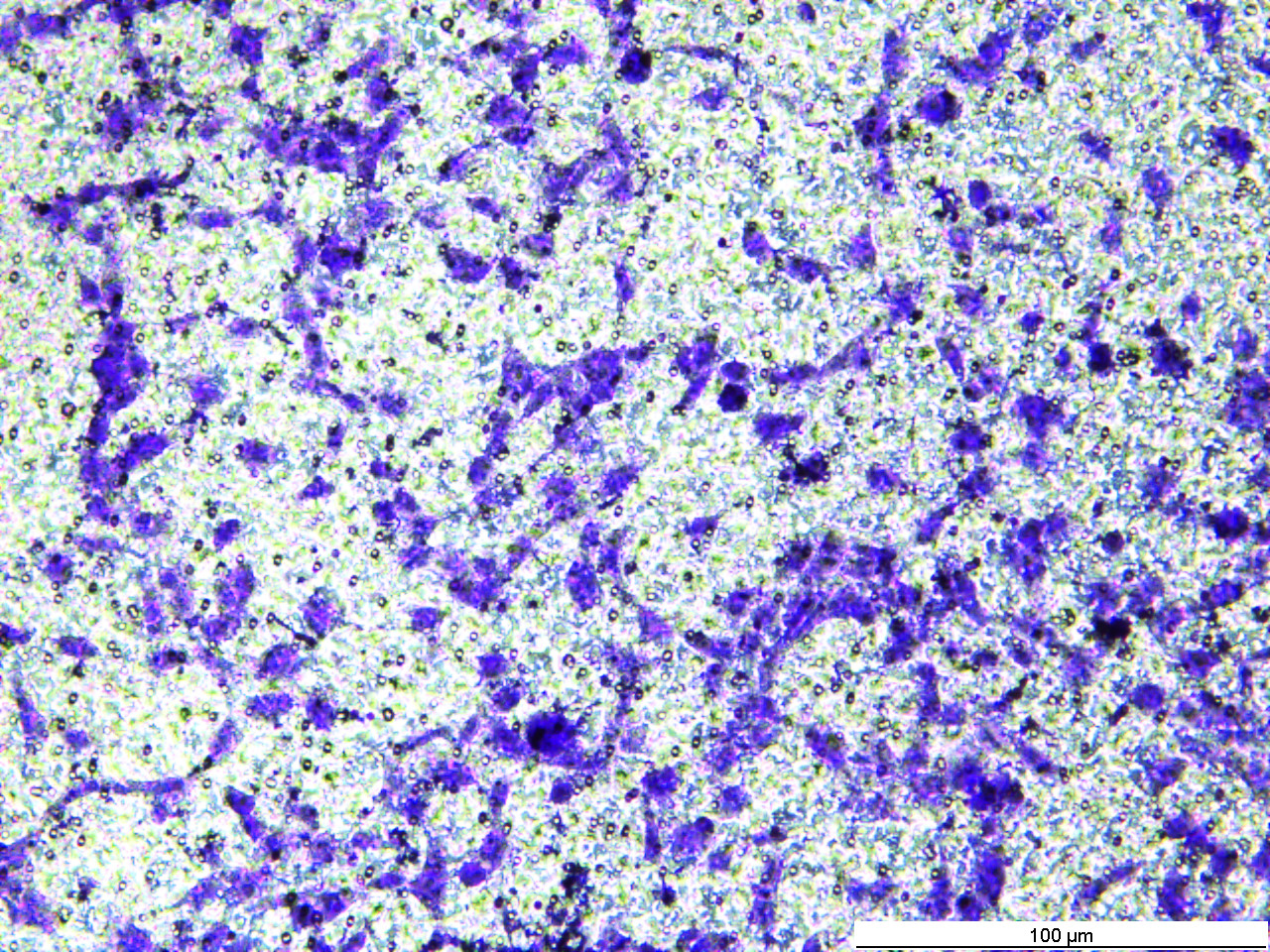

Supplement: Supplementary file 7 [file DataSheet_1.zip › Raw Data/microscopy images/Figure7G/Figure7G 786O-sh2.jpg]

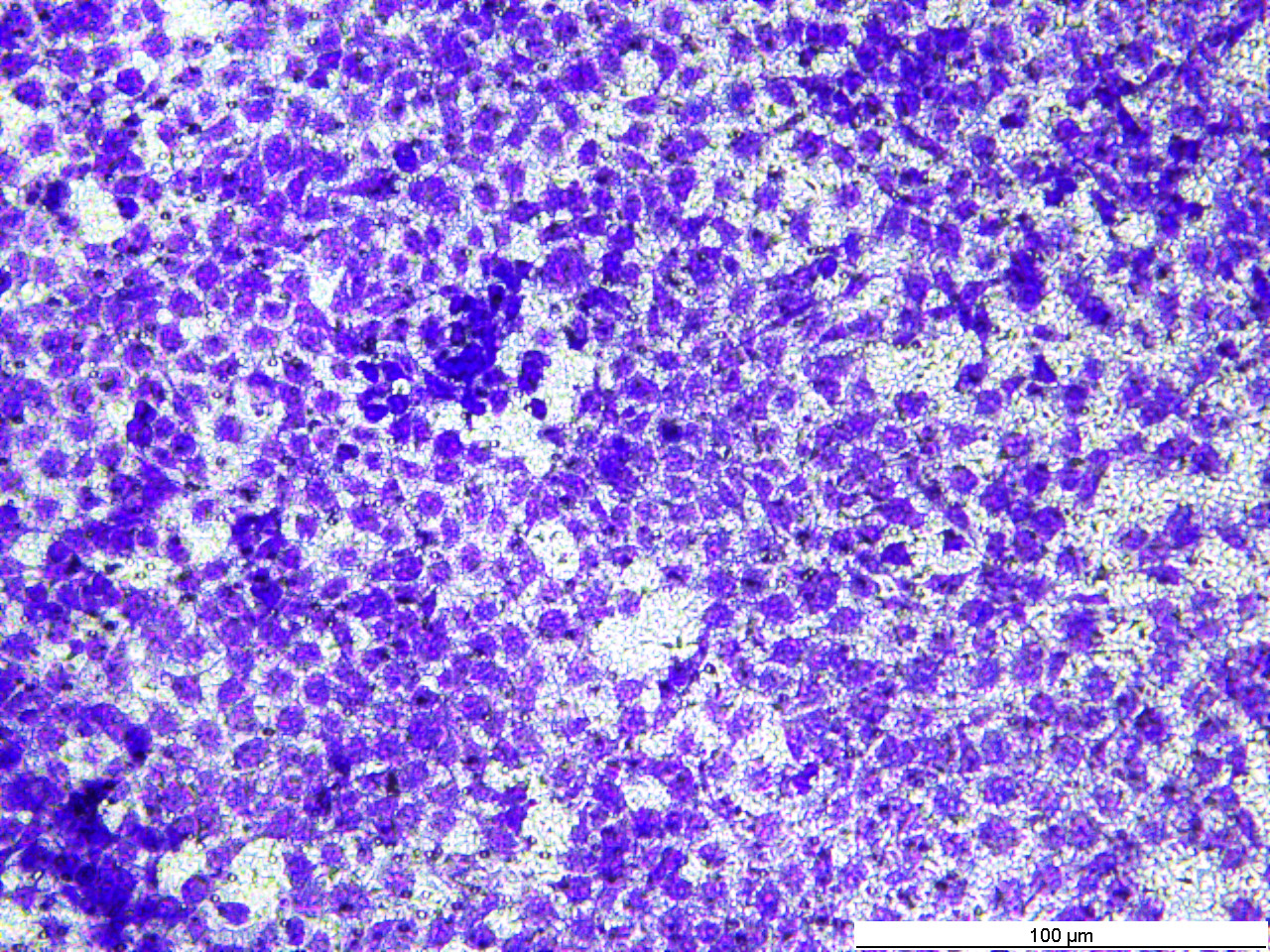

Supplement: Supplementary file 7 [file DataSheet_1.zip › Raw Data/microscopy images/Figure7G/Figure7G OS-NC.jpg]

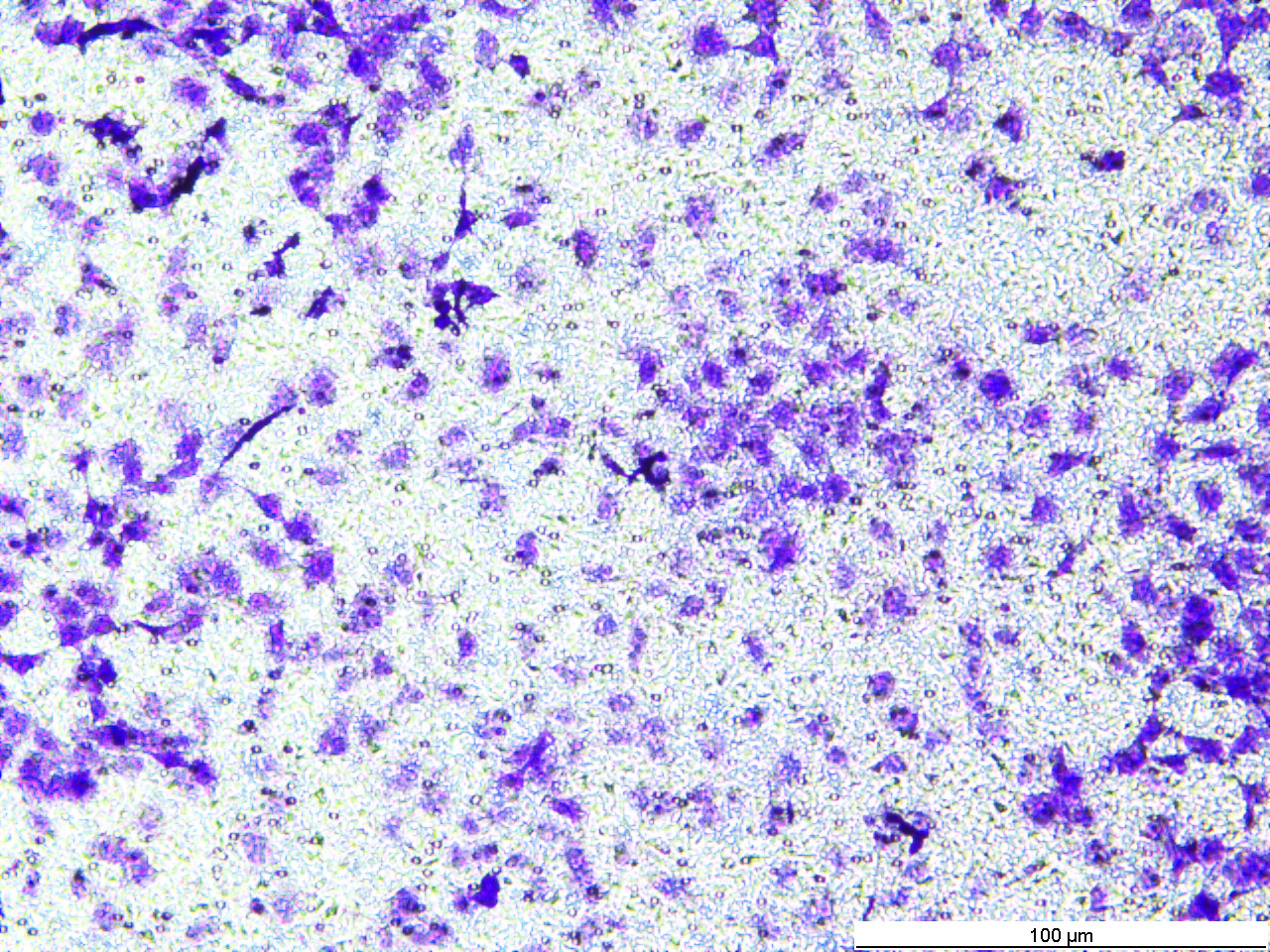

Supplement: Supplementary file 7 [file DataSheet_1.zip › Raw Data/microscopy images/Figure7G/Figure7G OS-sh1.jpg]

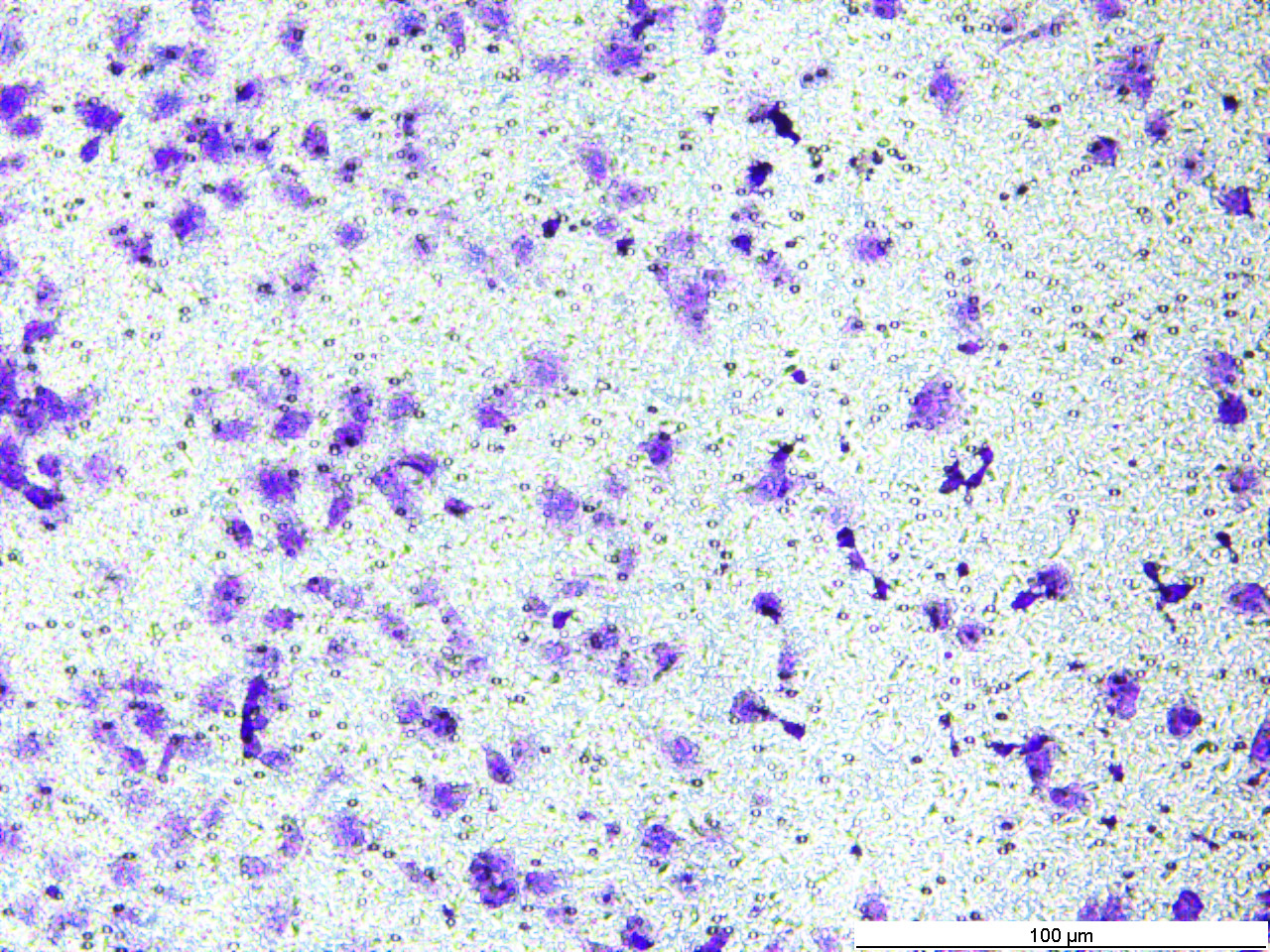

Supplement: Supplementary file 7 [file DataSheet_1.zip › Raw Data/microscopy images/Figure7G/Figure7G OS-sh2.jpg]

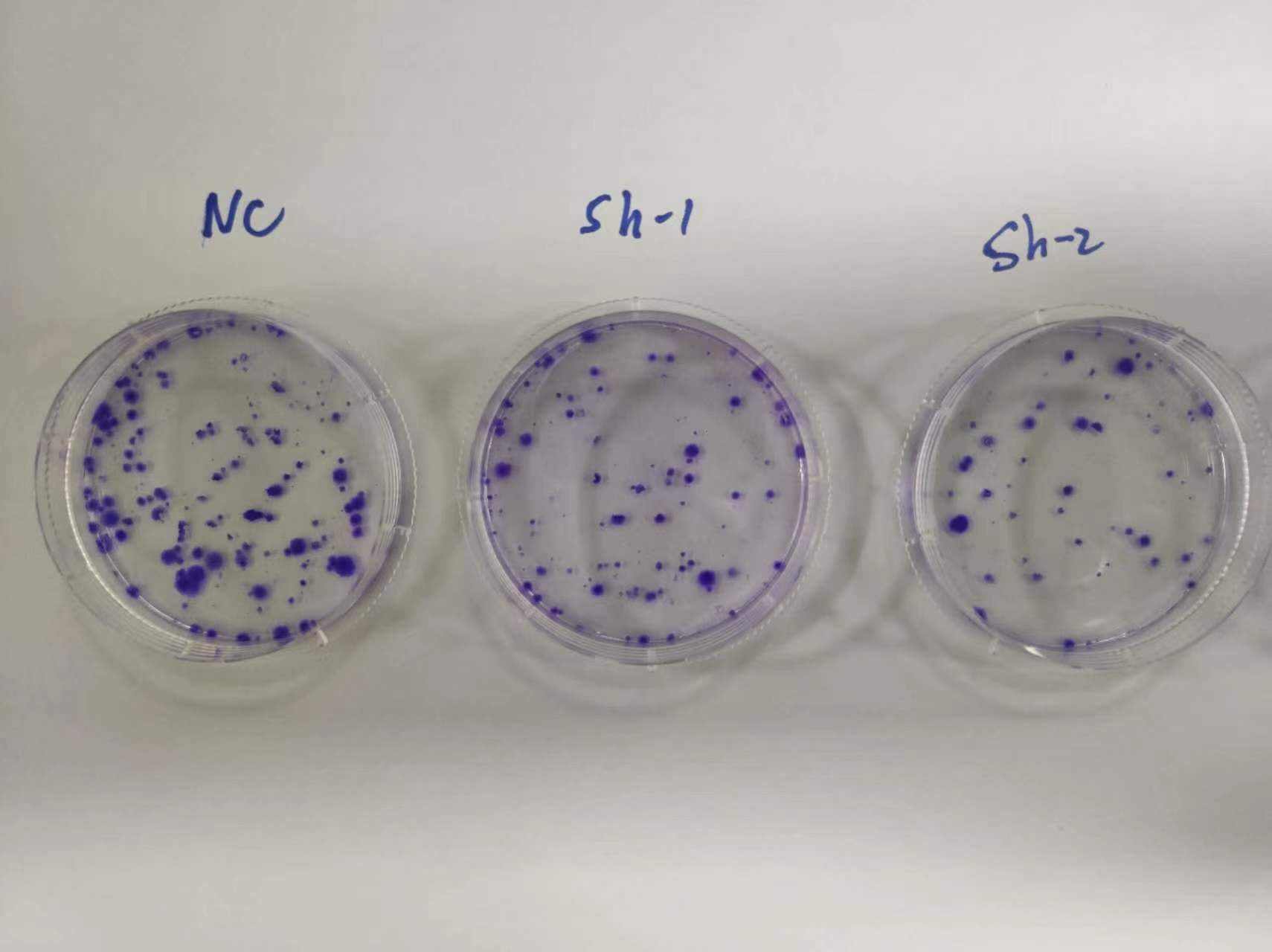

Supplement: Supplementary file 7 [file DataSheet_1.zip › Raw Data/microscopy images/Figure8E/5637-clones.jpg]

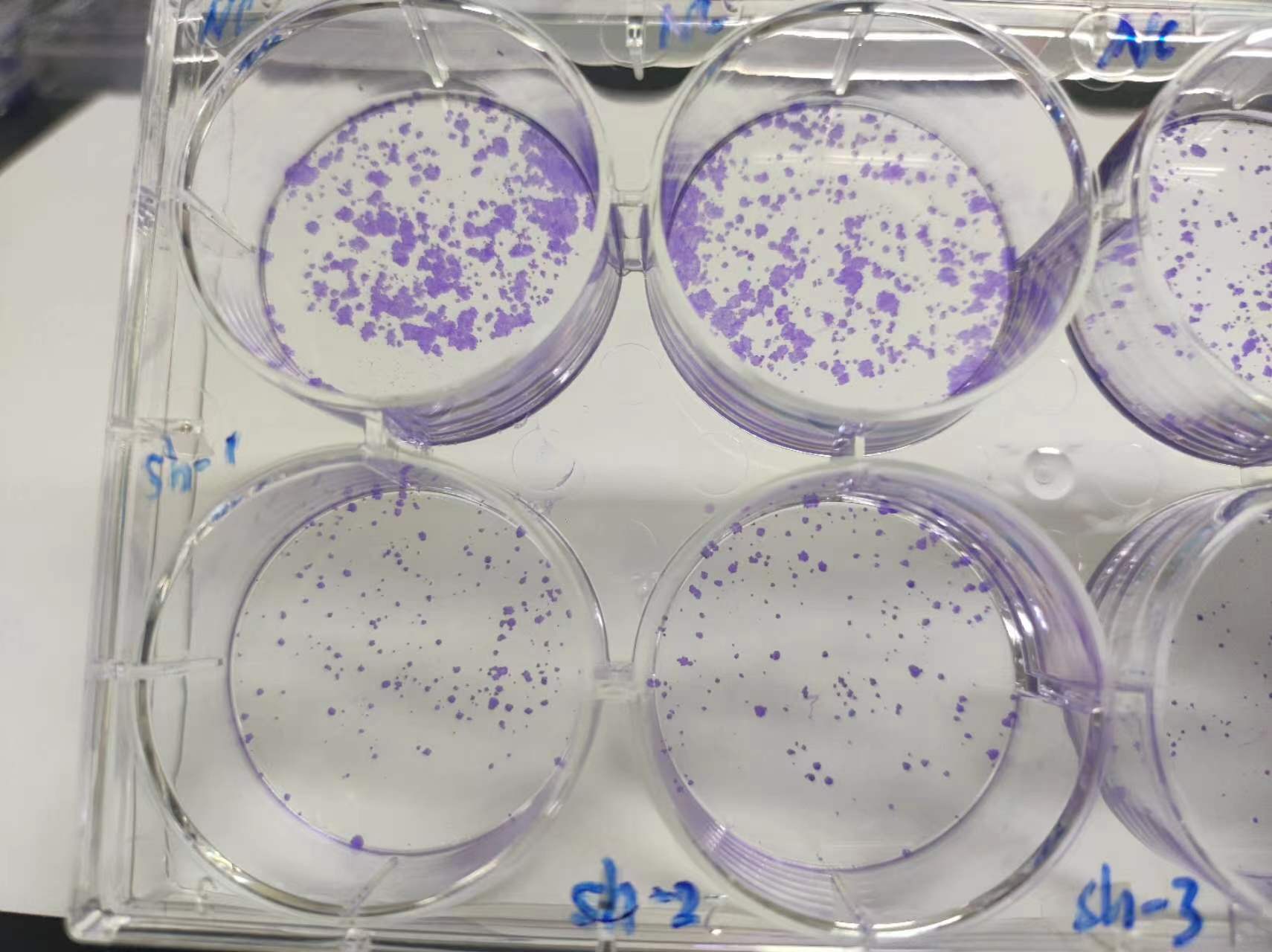

Supplement: Supplementary file 7 [file DataSheet_1.zip › Raw Data/microscopy images/Figure8E/T24-clones.jpg]

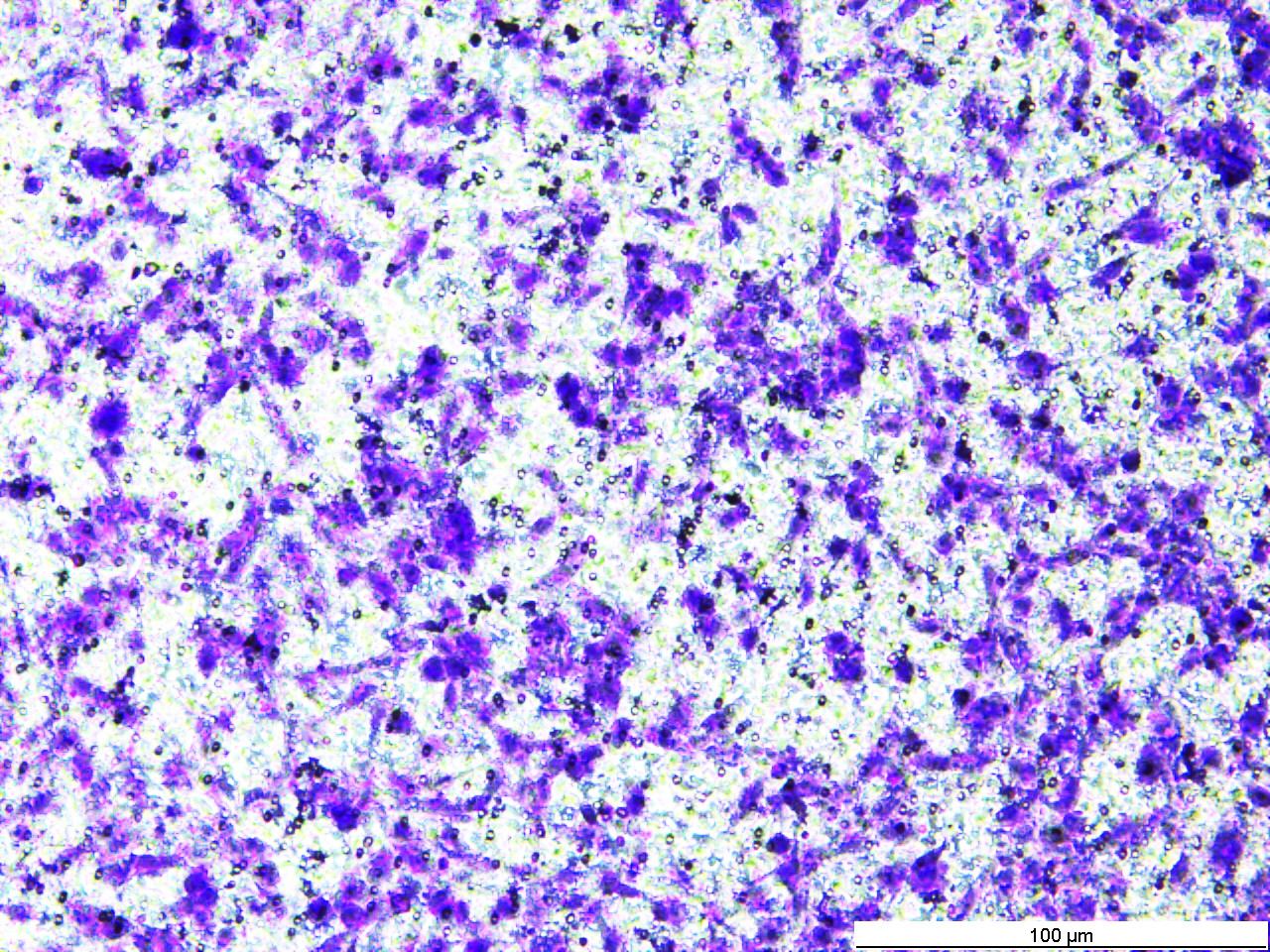

Supplement: Supplementary file 7 [file DataSheet_1.zip › Raw Data/microscopy images/Figure8G/Figure8G 5637-NC.jpg]

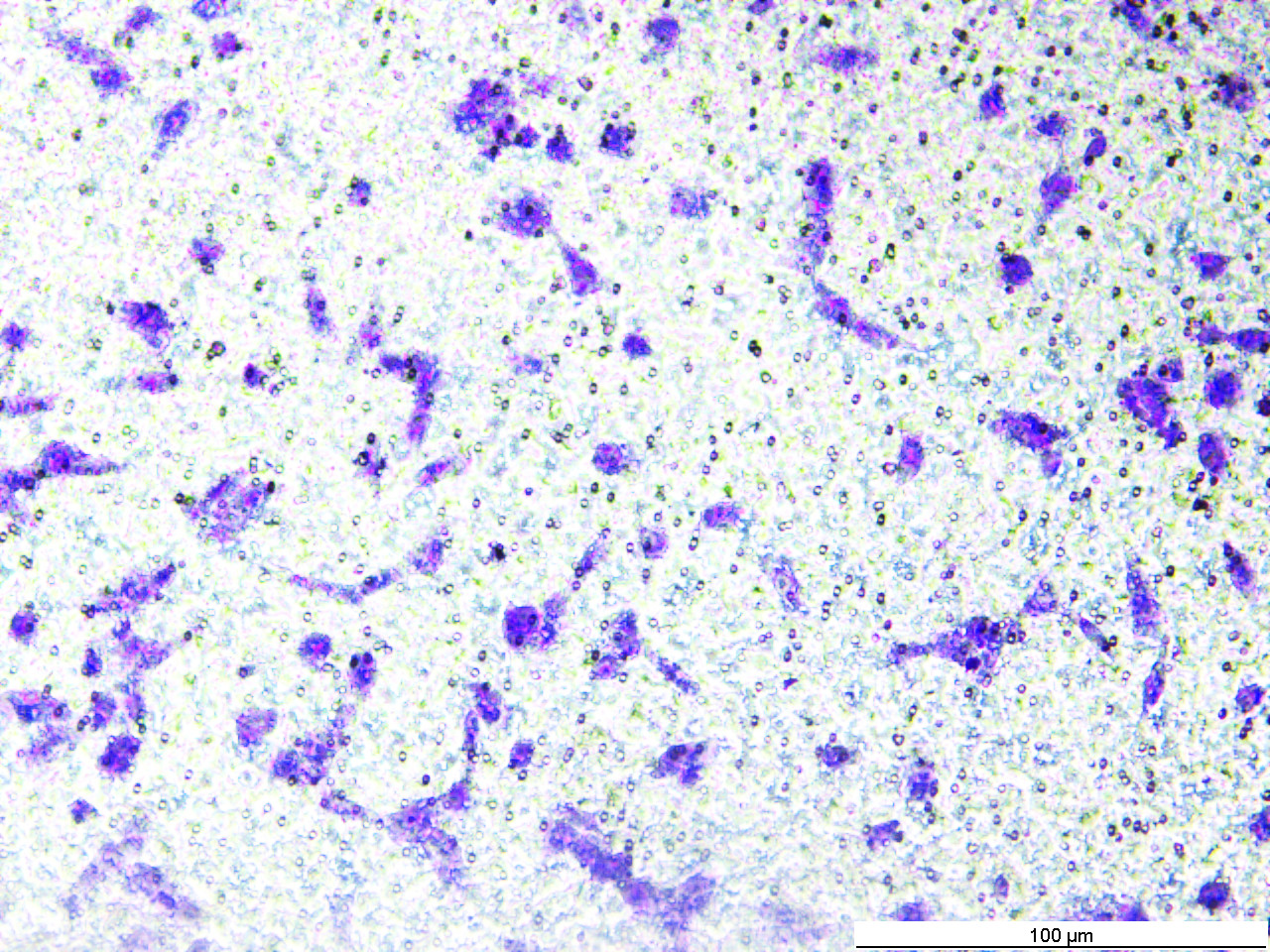

Supplement: Supplementary file 7 [file DataSheet_1.zip › Raw Data/microscopy images/Figure8G/Figure8G 5637-sh1.jpg]

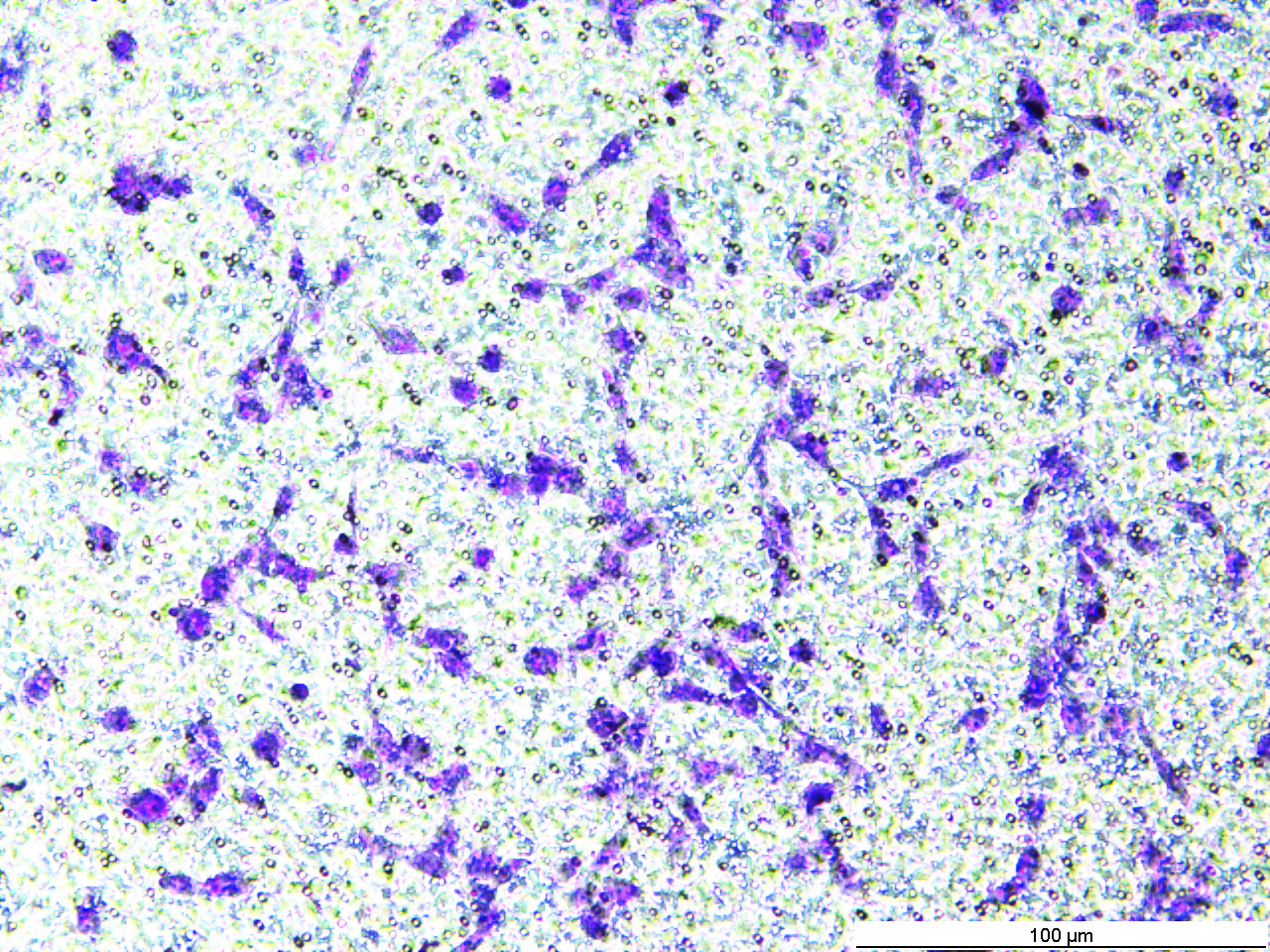

Supplement: Supplementary file 7 [file DataSheet_1.zip › Raw Data/microscopy images/Figure8G/Figure8G 5637-sh2.jpg]

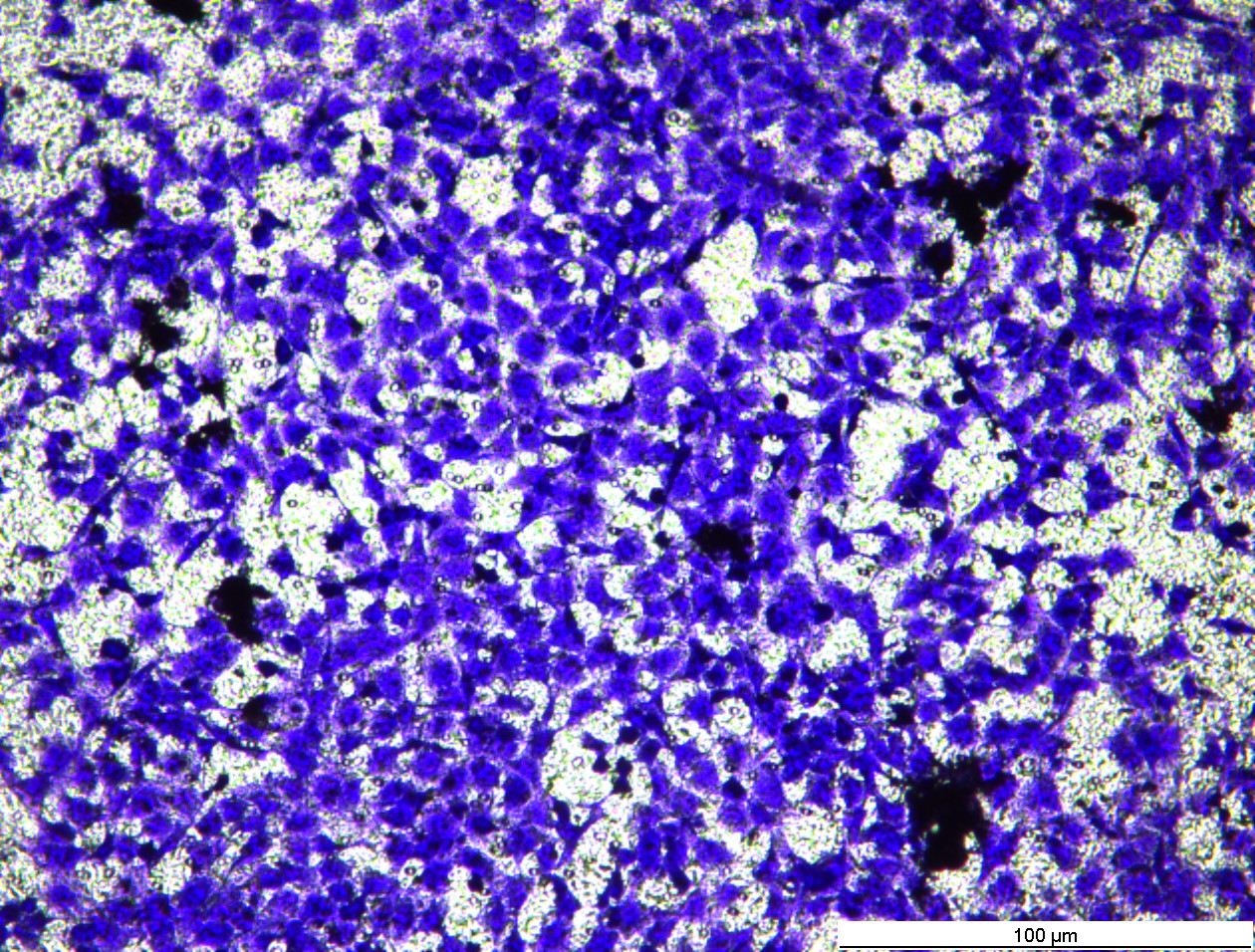

Supplement: Supplementary file 7 [file DataSheet_1.zip › Raw Data/microscopy images/Figure8G/Figure8G T24-NC.jpg]

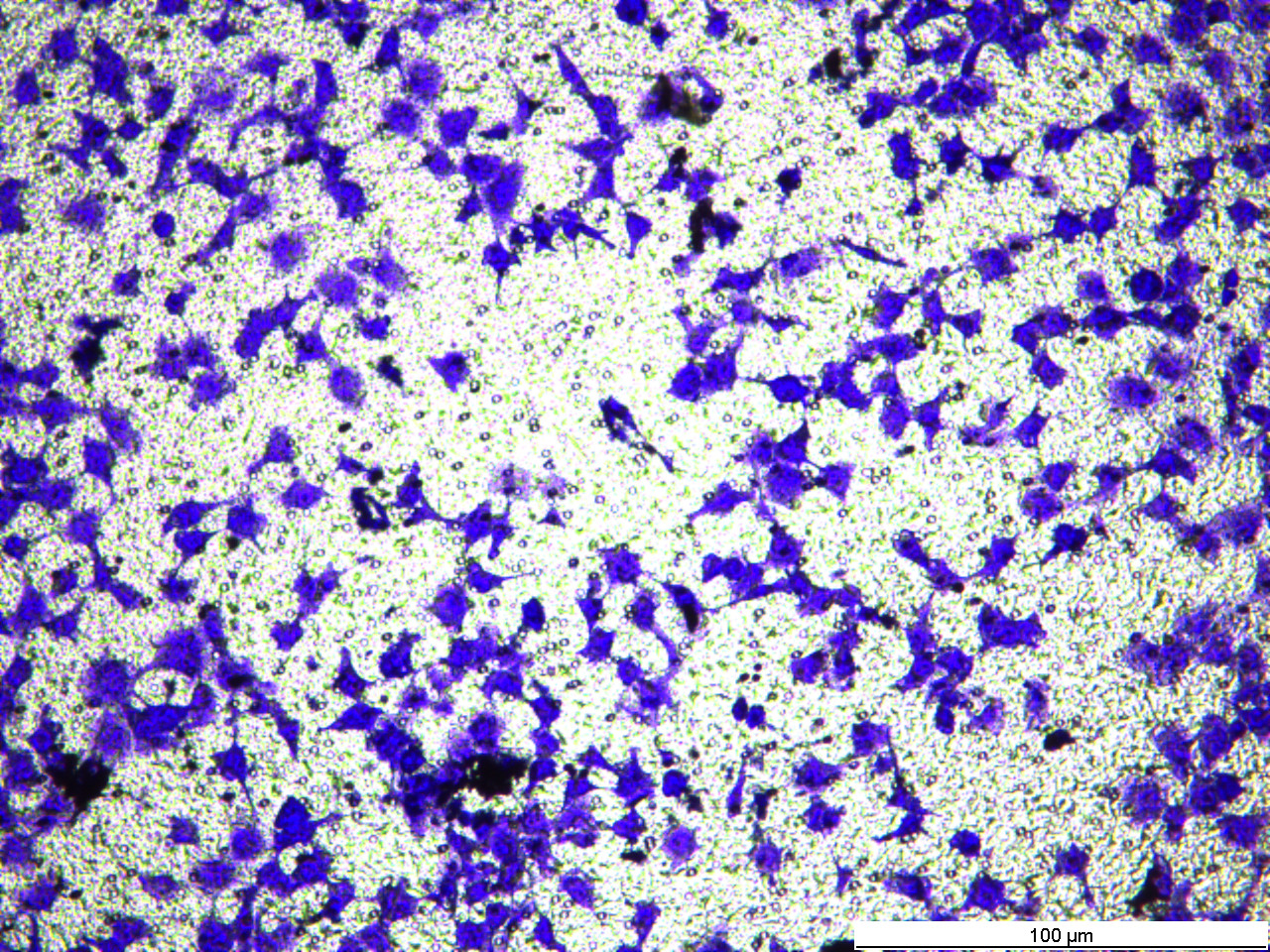

Supplement: Supplementary file 7 [file DataSheet_1.zip › Raw Data/microscopy images/Figure8G/Figure8G T24-sh1.jpg]

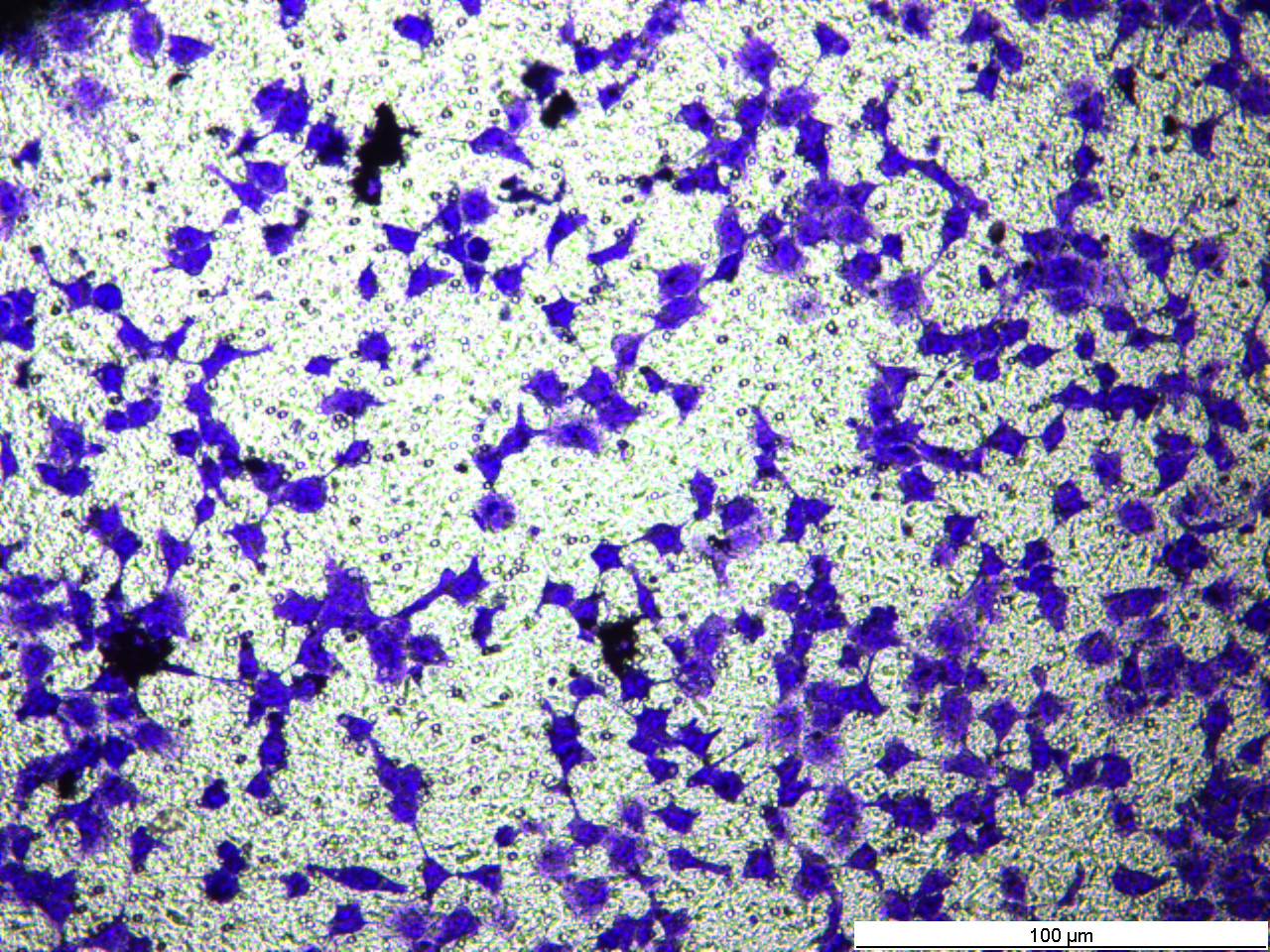

Supplement: Supplementary file 7 [file DataSheet_1.zip › Raw Data/microscopy images/Figure8G/Figure8G T24-sh2.jpg]
